# Amino acid transporter LAT1 (SLC7A5) promotes metabolic rewiring in TNBC progression through the L-Trp/QPRT/NAD^+^ pathway

**DOI:** 10.1186/s13046-025-03446-z

**Published:** 2025-07-03

**Authors:** Margot Y. Fedoroff, Lei Zhao, Shaomin Wang, Leili Saeednejad Zanjani, Alok Bhushan, Haifeng Yang, Karen M. Bussard, Stephen C. Peiper, Jun He

**Affiliations:** 1 Department of Pathology and Genomic Medicine, Sidney Kimmel Cancer Center, Thomas Jefferson University, Philadelphia, PA USA; 2 Department of Pharmaceutical Sciences, Jefferson College of Pharmacy, Thomas Jefferson University, Philadelphia, PA USA; 3 Department of Pharmacology, Physiology & Cancer Biology, Sidney Kimmel Cancer Center, Thomas Jefferson University, Philadelphia, PA USA

**Keywords:** LAT1 (SLC7A5), L-Tryptophan, TNBC, Glycolysis, NAD +/NADH, QPRT, LDHA, PKM2

## Abstract

**Background:**

Cancer cells uptake excessive nutrients by expressing higher levels of glucose and/or amino acid transporters to meet their increased energy demands. L-type amino acid transporter 1 (LAT1), is regarded as a cancer-specific transporter for the uptake of large neutral amino acids such as L-tryptophan. However, the mechanism by which LAT1 rewires cellular metabolism to promote cancer progression and chemoresistance have not yet been investigated.

**Methods:**

The protein levels of LAT1, p-PKM2, and p-LDHA were determined in breast cancer tissue arrays by immunohistochemistry staining followed by survival analysis. The orthotopic breast cancer models in mice, syngeneic breast cancer models, and patient-derived xenograft (PDX) mouse models were used to study the effects of LAT1 inhibition in tumor growth and chemoresistance. Steady-state polar metabolite analysis was performed to profile changes in cellular metabolism by LC-MS. The pyruvate and lactate assays as well as the seahorse assay using LAT1 knockdown cells and control cells were conducted to evaluate cellular glycolytic activities.

**Results:**

The LAT1 protein levels were positively correlated with poor survival in triple-negative breast cancer (TNBC) patients. LAT1 silencing resulted in reduced TNBC cell viability, proliferation, migration, invasion in vitro, as well as tumor growth in vivo*.* The knockdown of LAT1 reduced glycolytic activities via activating PKM2 and LDHA, two key glycolytic enzymes essential for cancer cell growth. Mechanistically, we demonstrated that LAT1 promoted de novo NAD + synthesis by facilitating L-tryptophan uptake and upregulating quinolinate phosphoribosyltransferase (QPRT), the rate-limiting enzyme in this pathway. This resulted in an increased cytosolic NAD^+^/NADH ratio, which enhanced the phosphorylation of pyruvate kinase M2 (PKM2) and lactate dehydrogenase A (LDHA), thus promoting TNBC tumor progression. Notably, upregulation of this pathway was observed in primary cells from doxorubicin (Dox)-resistant TNBC patient-derived xenograft (PDX) tumors and in Dox-resistant MDA-MB-231 cells. LAT1 inhibition sensitized resistant cells to Dox-induced cytotoxicity while supplementation of L-Trp/NAD + partially reversed the enhanced sensitivity to Doxorubicin induced by LAT1 knockdown. Furthermore, treatment with a LAT1-specific inhibitor JPH203 synergistically enhanced the efficacy of doxorubicin in TNBC cells.

**Conclusion:**

These findings identify a novel role of LAT1 in promoting TNBC progression and chemo-resistance by amplifying the Warburg effect, positioning LAT1 as a promising therapeutic target for TNBC treatment.

**Supplementary Information:**

The online version contains supplementary material available at 10.1186/s13046-025-03446-z.

## Introduction

Triple-negative breast cancer (TNBC), which accounts for 10% to 15% of breast cancer cases, is recognized as the most fatal subtype due to its high aggressiveness and limited treatment options, among other factors [1]. While the pathological complete response (pCR) rate in TNBC patients receiving neoadjuvant chemotherapy is approximately 20%, the majority of TNBC patients experience significantly higher recurrence rates, shorter post-recurrence survival, and worse overall survival compared to those with non-TNBC [2], indicating a pressing need to identify novel druggable targets for treating TNBC.

In malignant cells, higher-than-usual amounts of nutrients such as sugars and amino acids are taken in to support rapid cell proliferation and increased energy demands. Not surprisingly, cancer cells achieve this by expressing higher levels of nutrient transporters that mediate uptake of glucose and amino acids to meet their survival needs. L-type amino acid transporter 1 (LAT1), also known as SCL7A5, mediates the influx of neutral essential amino acids EAA (leucine, isoleucine, phenylalanine, methionine, histidine, tryptophan, valine, and tyrosine) into cells in exchange for the efflux of intracellular substrates such as EAA or glutamine [3]. In addition to its physiological role during placenta development [4], LAT1 exhibits high cancer-specificity in its expression and is overexpressed in various cancers including lung, gastric, prostate, pancreatic and liver cancer [5–12]. Clinicopathological studies revealed a positive correlation between LAT1 expression in cancers and poor survival outcomes [13]. Regarding breast cancer, high expression of SLC7A5 mRNA and protein was associated with poor patient outcome and endocrine resistance in luminal-type breast cancer [12]. SLC7A5 promoted cell proliferation of MCF-7 breast cancer cells through activating AKT/mTORC1 signaling pathway [14]. However, there are fewer studies on the role of SLC7A5 in the development of TNBC, drug resistance or prognosis. Dysregulation of amino acid transporters leads to metabolic reprogramming, which changes intracellular amino acid levels, contributing to the pathogenesis of cancer [15]. However, it remains elusive whether and how LAT1 rewires the cellular metabolic programs to promote TNBC tumor cell proliferation. In this study, we investigated the role of LAT1 in TNBC using *in-vitro* models, syngenetic xenograft tumor models, and PDX mouse models. Our aim was to determine whether targeting LAT1 reduces tumor progression, enhances the efficacy of chemotherapy, and to elucidate the underlying mechanisms involved.

## Material and method

### Cell culture and chemicals

Human TNBC cell lines MDA-MB-231, MDA-MB-468, and BT-549 were purchased from the American Type Culture Collection (ATCC, USA). MDA-MB-231 cells were cultured in DMEM medium (Corning, USA) supplemented with 10% (v/v) FBS (Gibco, USA) and 1% (v/v) Penicillin/Streptomycin (Gibco). MDA-MB-468 cells were cultured in RPMI-1640 medium (Gibco) supplemented with 10% (v/v) FBS (Gibco) and 1% (v/v) Penicillin/Streptomycin (Gibco). BT-549 cells were cultured in RPMI-1640 medium (Gibco) supplemented with 10% FBS (Gibco), 1% Penicillin/Streptomycin (Gibco), and 0.023 U/mL insulin (Sigma-Aldrich, USA). Cells were maintained in a humidified atmosphere with 5% CO_2_ in a cell incubator. An amino acid-free DMEM medium was prepared by dissolving medium powder (US Biological, USA) in sterilized water. The pH was adjusted to the appropriate level, and the medium was sterilized by filtration. Cells were incubated in the amino acid-free medium overnight prior to conducting the experiments. The following chemicals were used in this study: β-Nicotinamide adenine dinucleotide hydrate (NADH, Cat. #M041, AK Sci., USA), doxorubicin hydrochloride (Dox, Cat. #D1515, Sigma-Aldrich), and JPH203 (Cat. #S8667, Selleck, USA). They were added to the cell culture media or used in animal studies at the specified doses and durations. The Dox-resistant MDA-MB-231 (MDA-MB-231R) cells were induced by continuous treatment with Dox over a period of 4 months, with the dose gradually increasing from 30 to 120 nM. Resistance to Dox was confirmed by comparing the IC_50_ values of Dox in the parental and MDA-MB-231R cells.

### Immunoblotting assay

Protein samples from tissues and cells were extracted and lysed with RIPA lysis buffer containing protease and phosphatase inhibitors (Cat. #A32959, Thermo Scientific, USA). Whole-cell lysates were sonicated and centrifuged at 13,000 × g for 5 min. The clear supernatants were collected, and the protein concentrations were quantified using the BCA assay (Cat. #23225, Thermo Fisher Scientific). Proteins were then separated using 10% SDS-PAGE and transferred to PVDF membranes (Cat. #1620177, Bio-Rad, USA). The membranes were then blocked with 5% fat-free milk and incubated with primary antibodies at 4 °C overnight. Subsequently, the membranes were hybridized with HRP-conjugated secondary antibodies specific to the host species of the primary antibodies, and the protein bands were visualized using ECL (Cat. #34580, Thermo Scientific) and imaged using the ChemiDoc Imaging System (Bio-Rad). The primary antibodies used in this study include: LAT1 Rabbit Antibody (1:1000, #5347, Cell Signaling Technology, USA); Phospho-PKM2 (Tyr105) Rabbit Antibody (1:1000, #3827, Cell Signaling); PKM2 Rabbit Antibody (1:1000, #4053, Cell Signaling); LDHA Rabbit Antibody (1:1000, #3582, Cell Signaling); Phospho-LDHA (Tyr10) Rabbit Antibody (1:1000, #PA5-105445, Thermo Scientific); QPRT Rabbit Antibody (1:1000, #25174-1-AP, Protein Tech., USA); β-actin Mouse Antibody (1:2000, #sc-47778, Santa Cruz Biotechnology, USA).

### Cell migration and invasion assays

Cell migration was determined using 24-well transwell plates with 8 µm pores (Cat. #3422, Corning, USA). Cells were seeded in the upper chamber at a density of 5 × 10^4^/well in 200 µL medium without FBS. The bottom compartment was filled with 600 µL complete medium containing 10% FBS. After a 24-h incubation, a cotton swab was used to remove non-migrated cells from the upper side of the transwell cassette. The cells were then fixed with 70% ethanol and stained with 0.1% crystal violet solution. The number of migrated cells through each transwell chamber was counted under a microscope in five randomly selected fields. For the invasion assay, transwell inserts with 8 µm pore sizes (Corning) were coated with Matrigel (Cat. #354,230, Corning) to mimic the extracellular matrix. Approximately 2.5 × 10^4^ cells were seeded into the upper chamber in 200 µL of serum-free medium, while the lower chamber was filled with 600 µL of medium containing 10% FBS as a chemoattractant. After 24 h of incubation at 37 °C, non-invaded cells were removed from the upper surface of the membrane using a cotton swab. The invaded cells on the lower surface were then fixed with 70% ethanol, stained with 0.1% crystal violet, and counted under a microscope in five randomly selected fields.

### 3-(4,5-dimethylthiazol-2-yl)−2,5-diphenyl tetrazolium bromide (MTT) assay

TNBC cells were seeded in 96-well plates at a density of 3,000 cells per well, with 6 replicates per group. To measure cell viability, cells were starved for 6 h, and the culture medium was replaced with 100 µL of FBS-free medium supplemented with 10 µL of MTT reagent (5 mg/mL). The plates were then incubated for 2–4 h in a cell incubator, and absorbance was measured at 570 nm by a plate reader. For IC_50_ measurements, cells were seeded in 96-well plates at the same density and treated with Dox at various concentrations for 72 h. Cell viability in each well was assessed using the MTT assay according to the standard protocol, and the IC_50_ value was subsequently calculated.

### Bromodeoxyuridine (BrdU) assay

Cell proliferation was measured using the BrdU Cell Proliferation Colorimetric Assay Kit (Cat. #2750, Sigma-Aldrich) according to the manufacturer’s instructions. Briefly, cells were seeded in 96-well plates at 2 × 10^4^ cells/well and treated according to the experimental plan. For the BrdU assay, the BrdU stock solution was diluted 1:500 in a culture medium. Then, 20 µL of the diluted BrdU reagent was added to each well, except for the background control. The plates were incubated overnight for better BrdU incorporation. Cells were then fixed, and the DNA was denatured using the Fixing Solution (provided in the kit). BrdU incorporation was detected using an anti-BrdU monoclonal antibody (provided in the kit). Finally, the absorbance was measured at 450 nm using a plate reader to quantify BrdU incorporation in the DNA, which reflects the proliferation rate of the cells.

Immunofluorescent staining (IF) against BrdU was performed according to the manufacturer’s protocol. Briefly, cells were grown on coverslips, treated overnight with 10 µM BrdU (Cat. #ab142567, Abcam, USA), and then fixed with 4% paraformaldehyde at room temperature for 10 min. After acid hydrolysis and membrane permeabilization, the cells were blocked with 1% BSA solution and then incubated overnight with Alexa Fluor® 647-conjugated anti-BrdU antibody (Cat. #ab220075, Abcam). The cells were mounted with ProLong™ Diamond Antifade Mountant with DAPI (Cat. #P36962, Thermo Scientific) and imaged using a fluorescent microscope (Eclipse Ci, Nikon, USA).

### Oxidized nicotinamide adenine dinucleotide (NAD^+^)/NADH ratio assay

The cytosolic NAD^+^/NADH ratio was assessed using the NAD/NADH Quantification Kit (Cat. #MAK037, Sigma-Aldrich) following the manufacturer’s instructions. Briefly, 2 × 10^5^ cells were washed with cold PBS and then suspended in 400 µL of NADH/NAD Extraction Buffer. The cells were subjected to two repetitive freeze-thaw cycles on dry ice, and the cell lysates were then centrifuged at 13,000 X g for 10 min at 4 °C to remove insoluble material. The samples were deproteinized using a 10 kDa cut-off spin filter (Cat. #MRCRPT010, Millipore, USA) to remove enzymes that consume NADH rapidly. For total NAD detection, up to 50 µL of extracted samples were transferred in triplicate into a 96-well plate. For the NADH assay, an aliquot of each sample was heated at 60 °C for 30 min to decompose NAD⁺, leaving only NADH in the sample. Then, up to 50 µL of the heated samples were transferred in triplicate into a 96-well plate for NADH measurement. For both assays, 100 µL of the Master Reaction Mix, composed of NAD Cycling Buffer and Enzyme Mix, was added to each well. The reactions were incubated for 5 min at room temperature, and then 10 µL of NADH Developer was added into each well, followed by incubation for 2 h at room temperature. The absorbance at 450 nm was measured with a plate reader. The amount of NAD⁺ was determined by subtracting NADH from the total NAD. The ratio of NAD⁺ to NADH in a sample was calculated as (total NAD - NADH)/NADH.

### Pyruvate and lactate assays

Cytosolic pyruvate and lactate concentrations were measured using the Pyruvate Assay Kit (Cat. #MAK071, Sigma-Aldrich) and the Lactate Assay Kit (Cat. #MAK064, Sigma-Aldrich) following the manufacturers’ instructions. Briefly, 2 × 10^6^ cells were homogenized in the provided assay buffer for each assay, and each sample had at least three replicates. For each reaction, 50 µL of the sample was added to a 96-well plate, followed by the addition of 50 µL of Master Reaction Mix containing the pyruvate/lactate probe and pyruvate/lactate enzymes to each well. The reaction was incubated for 30 min at room temperature. Absorbance at 570 nm was measured, and the pyruvate or lactate concentrations in each sample were calculated based on the OD values of the corresponding wells.

### Annexin V/PI double staining flow cytometry assay

Cell apoptosis was measured using the FITC Annexin V Apoptosis Detection Kit with PI (Cat. #640914, BioLegend, USA), following the manufacturer’s protocol. To perform the assay, cells were washed twice with cold Cell Staining Buffer and then resuspended in Annexin V Binding Buffer at a concentration of 1 × 10^6^ cells/mL. A 100 µL aliquot of the cell suspension was transferred to a 1.5 mL tube, and 5 µL of FITC-conjugated Annexin V was added. The cells were gently vortexed and incubated for 30 min at room temperature in the dark. Then, 400 µL of Annexin V Binding Buffer was added to each tube, and 5 µL of PI was added to each tube before loading the sample. The fluorescence intensities of FITC and PI were acquired using the BD Symphony A5 (BD Biosciences, USA). Data were analyzed using the FlowJo software (FlowJo, LLC, USA).

### Steady-state polar metabolite analysis (LC-MS)

Steady-state polar metabolite analysis was performed to profile changes in cellular metabolism. This assay used two pairs of MDA-MB-231 derivative cells: shNC and shLAT1, as well as control and JPH203-treated cells. Briefly, cells were seeded onto 10 cm dishes, with five replicate dishes per group. When confluence reached 90%, cell number and protein concentration were determined from one of the dishes, while the remaining four dishes were used for the metabolomics assay. Each dish was washed twice with cold HBSS buffer containing Ca^2+^ and Mg^2+^, and then 1 mL of pre-made extraction solution (80% methanol, 20% water, with 1.5 µM internal standard mix) was added to each dish. Cells were scraped from the dishes using cell scrapers. The cell suspension was then transferred to a pre-chilled 1.5 mL centrifuge tube on dry ice. Samples were vortexed vigorously for 30 s and left on dry ice for at least 30 min before pelleting cell debris by centrifugation. After centrifugation, the supernatant was transferred to a new pre-chilled tube on dry ice. Repeat the centrifugation to ensure complete removal of cell debris, then transfer the supernatant to a cryovial (Cat. #V4632, Sigma-Aldrich). Samples were stored at −80 °C and then shipped to the Wistar LC-MS Core Facility on dry ice. Metabolite profiling was performed using a Q Exactive HF-X mass spectrometer coupled to a Vanquish LC System (Thermo Scientific). Data were acquired in both positive and negative ion modes with polarity switching on the mass spectrometer at the Wistar facility.

### Seahorse assay

Cells were seeded onto XF miniplates (Agilent Technologies, USA) at a density of 1.5 × 10^4^ cells per well in a growth medium. Sterile PBS buffer (400 µL) was added to the moats around the XF miniplates and to the sensor cartridges. The miniplates were kept in a regular cell culture incubator, while the sensor cartridge was stored overnight in a non-CO_2_ incubator. On the assay day, the assay medium was prepared and adjusted pH to 7.4. Cells were washed with the warm assay medium and then put in a non-CO2 incubator at 37 °C for 1 h. The sensor cartridge injection ports were loaded with glucose (final concentration 10 mM), oligomycin (final concentration 1 μM), and 2-DG (final concentration 50 mM), and then the cartridge was placed onto the XFp Analyzer for calibration. The XFp cell plate was then put into the analyzer and assayed. The values were normalized by the protein concentration for each well. The XF Report Generator was used for data analysis.

### Immunohistochemistry (IHC) staining

Human breast cancer tissue microarray slides (CHTN_BrCaStg1, University of Virginia, USA) and human normal/cancer tissue microarray slides (Cat. #HuCAT071, HuFPT072, HuFPT151, and BRN04a, TissueArray LLC., USA) were deparaffinized followed by IHC using standard protocols. The primary antibodies used in this study include: LAT1(1:250 28670-1-AP, Protein Tech.), p-PKM2 (Tyr105) (1:100, #3827 Cell Signaling), and p-LDHA (Tyr10) (1:100, #PA5-105445, Thermo Scientific). The staining intensity for each protein was scored on a scale of 0 to 3, with 0 representing no staining, 1 for weak staining, 2 for moderate staining, and 3 for strong staining. The percentage of positive cells was quantified and scored on a scale of 0 to 4, where 0 indicates 0-5% positive cells, 1 for 6-25%, 2 for 26-50%, 3 for 51-75%, and 4 for > 75% positive cells. An IHC staining score for each protein was calculated by multiplying the staining intensity score by the percentage of positive cells score for each sample.

### LAT1 siRNA transfection

TNBC cells were plated in 6-well plates. When the cells reached 60-70% confluence, they were transfected with 100 nM ON-TARGETplus Human LAT1 siRNA SMARTPool (Cat. #L-004953-01-0010, Horizon Discovery, USA) using the jetPRIME transfection reagent (Cat. #89129-920, VWR, USA) according to the manufacturer’s protocol to knock down LAT1. Cells transfected with the ON-TARGETplus Non-targeting Control Pool (siNC, Cat. #D-001810-10-20, Horizon Discovery) were used as controls. The cells were incubated for 72 h before RNA and protein samples were harvested.

### Lentiviral-mediated shRNA knockdown of LAT1

To stably knock down LAT1 in MDA-MB-231cells, we used four shRNA constructs targeting different sites on LAT1 mRNA, which were cloned into the pGFP-C-shLenti lentiviral vector (Cat. #TL309279V, Origene, USA). A scrambled shRNA control (Cat. #TR30021V, Origene) was included as a negative control (shNC). Lentivirus harboring the shRNA constructs was packaged in 293T cells following the standard protocol. The virus-containing supernatants were collected 48 h after transfection, and MDA-MB-231 cells were transduced with the virus in the presence of 8.0 μg/mL polybrene. After transduction, cells were treated with 1.0 µg/mL puromycin to select for cells stably expressing the shRNAs. LAT1 downregulation was confirmed by Western blots.

### Orthotopic xenograft, syngeneic, and patient-derived xenograft (PDX) mouse models

For the tumor growth assay, 6-week-old female nude mice (*N* = 10 per group) were anesthetized to ensure they are immobile and do not experience pain during the procedure. The area around the mammary fat pad was cleaned with an antiseptic solution to minimize the risk of infection. A small incision was made between the fourth nipple and the midline, and a volume of approximately 100 µL containing 1 × 10^6^ shNC or shLAT1 expressing MDA-MB-231 cells were injected into the mammary fat pads. The site of injection was then closed and carefully sutured using a needle driver. After injection, the mice were monitored for signs of distress or infection. [16]. Tumor growth was monitored by measuring tumor volume every three days. Tumor volume was calculated using the formula: (length × width^2^)/2. Animal body weight was also measured every three days. After 21 days post-injection, the mice were euthanized, and tumors were harvested for further analysis.

To evaluate the effect of JPH203 on tumor growth, 6-week-old female C57BL/6 mice (5 mice per group) were anesthetized to ensure they are immobile and do not experience pain during the procedure. The area around the mammary fat pad was cleaned with an antiseptic solution to minimize the risk of infection. A small incision was made between the fourth nipple and the midline, and a volume of approximately 100 µL containing 5 × 10^5^ PY8119 cells expressing firefly luciferase were carefully injected into the mammary fat pad. The site of injection was then closed and carefully sutured using a needle driver. After injection, the mice were monitored for signs of distress or infection. [16] Mice were treated with either vehicle control or 15 mg/kg of JPH203 via subcutaneous injection a week after the implantation, five times per week. The body weight and tumor volume of the mice were measured every three days after tumor formation, as mentioned above. To visualize the size of xenograft tumors, mice were intraperitoneally injected with 150 mg/kg VivoGlo Luciferin (Cat. # P1042, Promega, USA). Whole-body bioluminescence images were acquired using the IVIS Lumina III in vivo imaging system (PerkinElmer, USA).

NSG mice bearing TNBC PDX tumors (TM00090, Jackson Lab, USA) were treated either with vehicle control or 0.6 mg/kg Dox twice a week, starting after visible tumor formation. Mice were sacrificed when the tumors reached the size of 1cm^3^, and the tumors were harvested, minced, and then subcutaneously implanted into new mice to generate the next generation of tumors for continuous treatment. The Dox dosage gradually increased at each generation from 0.6 mg/kg to 1.8 mg/kg. By the end of the fourth generation, tumors were harvested and processed for dissociation into primary cells using enzymatic digestion. All mice were housed and cared for in the animal care facility at Thomas Jefferson University. The study was approved by the Institutional Animal Care and Use Committee (IACUC) of Thomas Jefferson University. All procedures were conducted in accordance with federal and institutional guidelines for animal care and use.

### Statistical analysis

Statistical analysis was performed using GraphPad Prism 9 software (GraphPad, USA). Data are presented as mean ± standard deviation (SD) from at least three independent experiments and analyzed using a two-tailed Student’s t-test. Breast cancer datasets were retrieved from TCGA and subjected to gene expression, survival curve, and Gene Set Enrichment Analysis (GSEA). The Survival data were analyzed using the Kaplan–Meier method in SPSS Software (IBM, USA). The log-rank test was used to determine the significance between the survival curves. The GSEA analysis was conducted using the GSEA software (v. 4.3.3, Broad Institute, USA) with default settings. Differences were considered significant at a *P* value of ≤ 0.05.

## Results

### LAT1 is upregulated in BC patients with poor prognosis, especially in TNBC subtype

**Fig. 1:**
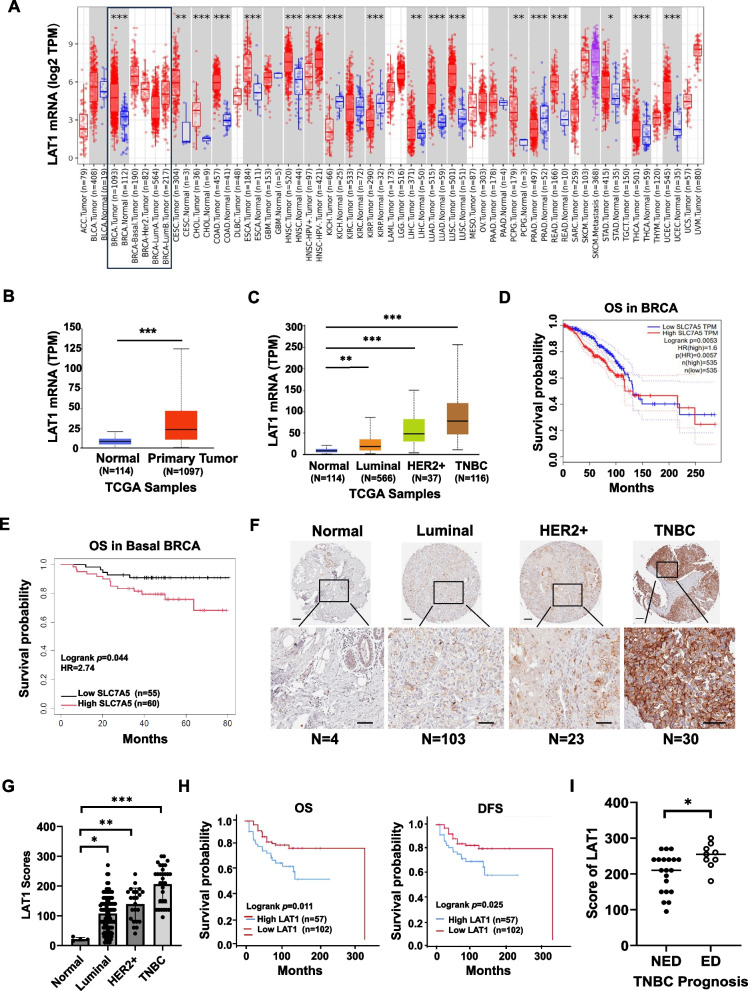
LAT1 is upregulated in TNBC and associated with poor prognosis. **A** A pan-cancer analysis of LAT1 mRNA expression across various cancer types and their corresponding normal tissues using the TCGA database. **B-E** LAT1 mRNA levels and clinical data for breast cancer (BC) were retrieved from the TCGA database. **B** LAT1 levels in normal breast tissues and BC tissues. **C** LAT1 levels in three subtypes of BC—luminal, HER2 +, and TNBC—compared to normal breast tissues. **D** Kaplan–Meier plot of overall survival (OS) in BC patients with high or low LAT1 expression. Survival curves were compared using the log-rank test. **E** Kaplan–Meier analysis of OS in the basal subtype of BC with high or low LAT1 expression. Survival curves were compared using the log-rank test. **F** Human breast cancer tissue microarrays were stained for LAT1 protein expression using IHC in normal breast tissues and three subtypes of BC, including luminal, HER2 +, and TNBC. An IHC staining score for LAT1 was calculated for each sample based on both the staining intensity and the percentage of positive cells. Representative images are shown. Scale bar = 600 µm (Top); 300 µm (Inset). **G** Quantification of the IHC staining for LAT1. **H** A Kaplan–Meier plot of OS (left) and disease-free survival (DFS, right) was generated for BC patients with high or low LAT1 expression, stratified based on IHC staining scores. Survival curves were compared using the log-rank test. **I** The correlation analysis between LAT1 expressions and prognosis of TNBC patients. NED: No evidence of disease. ED: Evidence of disease. * *P* < 0.05; ** *P* < 0.01; *** *P* < 0.001

The TCGA data showed that LAT1 expression was upregulated in various cancers at mRNA levels compared to their corresponding normal tissues (Fig. 1A). Specifically, LAT1 expression was significantly elevated across all breast cancer subtypes, with the highest levels observed in basal-like breast cancer (Fig. 1B-C). However, no significant correlation was observed between LAT1 expression and the stage or nodal metastasis status of breast cancer (Suppl. Fig.S1A, B). The Kaplan–Meier analysis showed that higher LAT1 expression was associated with poor overall survival (OS) in all breast cancers (Fig. 1D) as well as in the basal-like subtype (Fig. 1E). Moreover, LAT1 protein expression levels were assessed in human breast cancer tissue arrays through IHC staining. Consistent with the TCGA data, LAT1 protein expression was significantly higher in luminal, HER2^+^, and TNBC breast cancer tissues relative to normal tissues, with TNBC showing the highest level of LAT1 (Fig. 1F, 1G). Higher LAT1 expression was associated with poor OS and disease-free survival (DFS) in all breast cancers, as determined by analyzing IHC staining scores and clinical data (Fig. 1H). Additionally, our correlation analysis of LAT1 expression and prognosis data revealed that patients with recurrent TNBC (evidence of disease, ED) had higher LAT1 expression than those with a favorable prognosis (no evidence of disease, NED) observed in follow-up period of 2 to 12 years post-diagnosis, further indicating that higher LAT1 levels are associated with worse outcomes (Fig. 1I). Overall, the TCGA and tissue array data showed that LAT1 is significantly upregulated in breast cancer, suggesting that LAT1 may play a role to the progression of breast cancers, particularly in TNBC.

### Inhibition of LAT1 suppresses TNBC cell proliferation, migration, and tumor growth

**Fig. 2:**
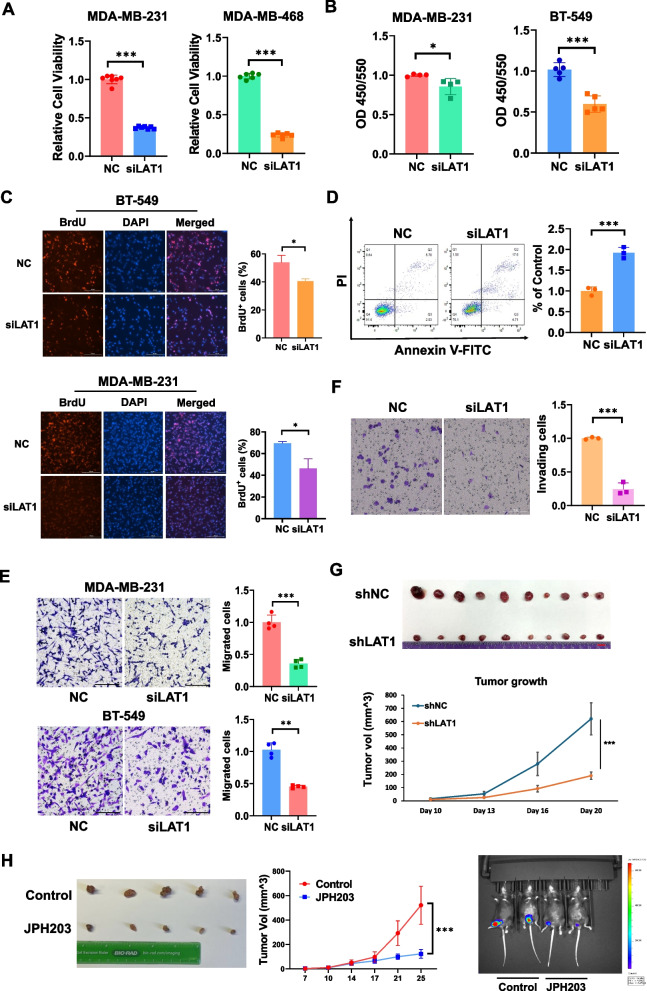
Inhibition of LAT1 suppresses TNBC cell viability, proliferation, migration, and in vivo tumor growth. **A-F** Cells were transiently transfected with 100 nM Smartpool LAT1 siRNA or a negative siRNA control (NC) for 72 h before the downstream assays. **A** MTT assay was performed to assess cell viability. **B** A colorimetric BrdU incorporation assay was performed to evaluate cell proliferation. **C** Immunofluorescent staining for BrdU was performed to assess cell proliferation. Left: Representative images. Right: Percentage of BrdU-positive cells. **D** Cell apoptosis was measured using Annexin V-FITC and PI double staining with flow cytometry. Left: Representative images. Right: Fold change in the rate of apoptotic cells. **E** A transwell migration assay was performed to assess cell migration ability. Left: Representative images. Right: Fold change in the number of migrated cells. Scale bar = 200 µm. **F** A transwell *in-vitro* invasion assay was conducted to assess cell invasion ability. Left: Representative images. Right: Fold change in the number of invading cells. Scale bar = 200 µm. **G** An orthotopic breast cancer model was established in nude mice using MDA-MB-231 control cells or LAT1 knockdown cells. The tumor images and growth curves were shown. *N* = 10 per group. Scale bar = 1 cm **H** A syngeneic breast cancer model was established in C57BL/6 mice using PY8119 murine TNBC cells. The mice were treated with either vehicle control or 15 mg/kg of JPH203 via subcutaneous injection five times per week for two weeks. Left: Representative images of tumors. Middle: Tumor growth curve. Right: In vivo bioluminescence imaging of the tumors. Three independent experiments were performed with a minimum of three biological replicates. * *P* < 0.05; ** *P* < 0.01; *** *P* < 0.001

To investigate the role of LAT1 in TNBC progression, we transiently knocked down LAT1 using siRNA in TNBC cell lines MDA-MB-231, MDA-MB-468, and BT-549 (Suppl.Fig.S2A). Silencing LAT1 resulted in approximately 60% and 75% reductions in cell viability in MDA-MB-231 and MDA-MB-468 cells, respectively, compared to scramble controls by MTT assays (Fig. 2A). Cell proliferation was further assessed using both colorimetric BrdU assays and immunofluorescent BrdU staining. The results showed that the amount of newly synthesized DNA, labeled by incorporated BrdU, was significantly decreased in LAT1 knockdown TNBC cells compared to the controls (Fig. 2B, C), indicating that the downregulation of LAT1 significantly inhibited TNBC proliferation. The effect of LAT1 depletion on TNBC cell apoptosis was evaluated in MDA-MB-231 cells using Annexin V-FITC/PI staining followed by flow cytometry. LAT1 knockdown resulted in a two-fold increase in apoptotic cells (Fig. 2D). We next assessed the impact of LAT1 downregulation on TNBC cell migration and invasion. Silencing LAT1 caused significantly reduced cell migration and invasion ability using transwell dishes and Matrigel coated transwell dishes respectively (Fig. 2E, F), and wound healing assays (Suppl. Fig.S2B) in TNBC cells. To determine whether the reductions of migration and invasion were directly caused by LAT1 silencing, we pretreated in LAT1 knockdown cells with mitomycin C to inhibit proliferation, followed by a transwell migration assay. We found the proliferation inhibition did not reverse migration reduction induced by LAT1 silencing, suggesting that the observed effects on migration and invasion are independent of proliferation changes (Suppl. Fig.2C). To validate our in vitro findings, we established stable LAT1 knockdown MDA-MB-231 cells using lentiviral LAT1 shRNA constructs (shLAT1), followed by puromycin selection. The control cells were transduced with a lentiviral expression vector (shNC). LAT1 downregulation was confirmed using immunoblotting (Suppl. Fig.S2D). Cells transfected with plasmid KD#2, which achieved superior LAT1 knockdown efficiency, were selected for subsequent experiments. We injected the LAT1 knockdown or control cells into the mammary pads of nude mice to evaluate tumor growth. Twenty days post-injection, we observed a significant reduction in tumor growth in the shLAT1 group compared to the control group (Fig. 2G). To further validate these findings, we established a syngeneic xenograft mouse model by orthotopically injecting luciferase-tagged murine TNBC PY8119 cells into the mammary pads of C57BL/6 mice. The mice were treated with either vehicle control or the LAT1 inhibitor JPH203 at a dose of 15 mg/kg, five times per week for two weeks. The JPH203-treated group exhibited significantly reduced tumor volume compared to the control group, as measured by tumor dimensions and bioluminescence images (Fig. 2H), without significant loss of body weight in mice (Suppl. Fig.S2E). Collectively, these results show that the inhibition of LAT1 leads to significant impairment of cell viability, proliferation, migration, invasion and tumor growth, as well as increased apoptosis, suggesting that LAT1 contributes to TNBC tumor growth and progression.

### LAT1 silencing rewires TNBC cell metabolism by reducing glycolytic activity

**Fig. 3:**
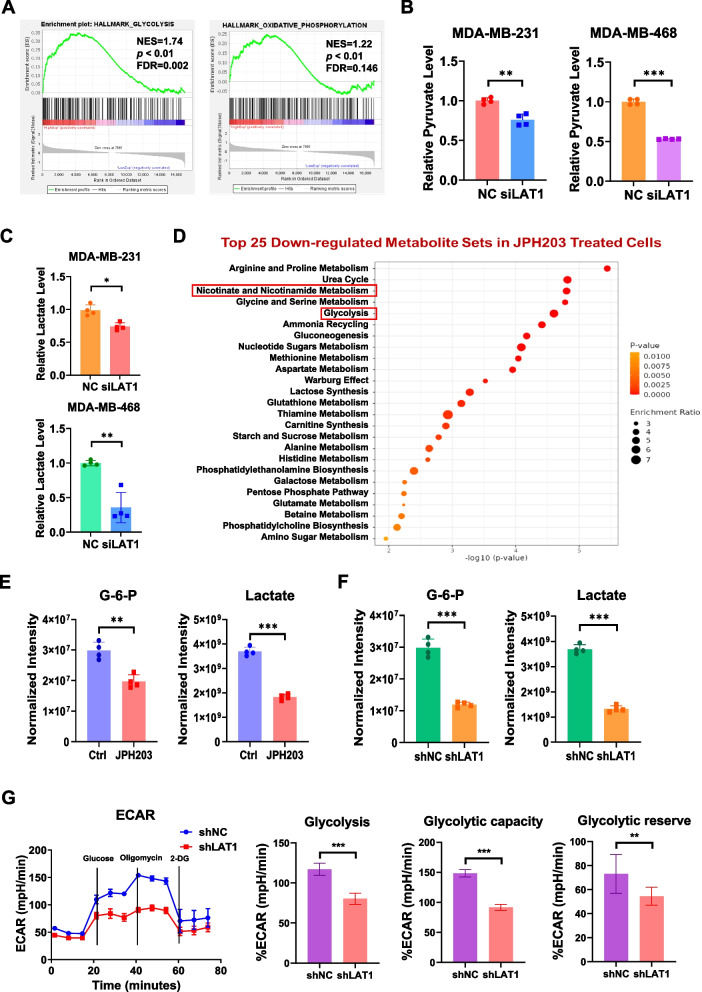
LAT1 silencing rewires TNBC cell metabolism by reducing glycolysis. **A** Differentially expressed genes were identified between BC patients with high and low LAT1 expression levels using TCGA data. GSEA was then performed on differential genes to identify enriched hallmark pathways. **B-C** Cells were transiently transfected with 100 nM Smartpool siLAT1 or a negative siRNA control (NC) for 72 h. The relative levels of cytosolic **B** pyruvate and **C** lactate were measured using the Pyruvate Assay Kit and Lactate Assay Kit, respectively, according to the manufacturer’s instructions. **D-F** Metabolites were profiled in shNC and shLAT1 MDA-MB-231 cells using an LC-MS-based assay. **D** Metabolite enrichment analysis was performed to analyze the differentially expressed metabolites using MetaboAnalyst software. The bar chart shows the enriched metabolic pathways in the differential metabolites. **E** The levels of G-6-P, and lactic acid in shNC and shLAT1 MDA-MB-231 cells were determined by the metabolomics assay. Peak intensities were normalized for comparison (*n* = 4). **F** MDA-MB-231 cells were treated with vehicle control or 10 µM JPH203 for 10 min. The levels of G-6-P, and lactic acid were measured by LC-MS assay. Peak intensities were normalized for comparison (*n* = 4). **G** A Seahorse assay was conducted to measure ECAR in shNC or shLAT1 MDA-MB-231 cells (*n* = 3). Three independent experiments were performed with a minimum of three biological replicates. * *P* < 0.05; ** *P* < 0.01; *** *P* < 0.001; ns. not significant

As a gatekeeper of the influx of large neutral essential amino acids, LAT1 plays a crucial role in maintaining an adequate amnio acid reservoir to support cancer cells [17]. However, the impact of LAT1 as an amino acid transporter on cancer metabolism remains unexplored. Differentially expressed genes were identified between breast cancer patients with high and low-to-medium LAT1 expression levels using TCGA data. GSEA was then performed on differential genes to identify enriched hallmark signatures. The results revealed that the glycolysis gene set was significantly enriched in the high LAT1 expression group (NES = 1.74, *p* < 0.01, FDR = 0.002). Although the oxidative phosphorylation gene set showed a positive enrichment score (NES = 1.22, *p* < 0.01),

it has an FDR of 0.146, which would not be considered as significant at the 0.05 threshold (Fig. 3A). To assess the role of LAT1 in anaerobic glycolysis, we knocked down LAT1 in TNBC cells using siLAT1 and observed a significant reduction in cellular pyruvate (Fig. 3B) and lactate (Fig. 3C) production in both MDA-MB-231 and MDA-MB-468 cells, indicating that silencing LAT1 inhibited glycolysis. To further investigate the mechanisms by which LAT1 regulates glycolysis, we profiled metabolites changes in response to LAT1 inhibition by performing LC-MS-based metabolomic analysis in shLAT1-expressing MDA-MB-231 cells, MDA-MB-231 cells treated with JPH203, and their corresponding control cells. Pathway enrichment analysis of metabolites revealed significant enrichment in the nicotinate and nicotinamide metabolism and glycolysis pathways in the JPH203-treated cells compared to the control cells (Fig. 3D, Suppl. Fig.S3A, B). Additionally, the LC-MS data showed that the levels of key glycolytic metabolites, including glucose-6-phosphate (G-6-P), and lactic acid, were significantly decreased in both JPH203-treated cells (Fig. 3E) and shLAT1-expressing cells (Fig. 3F), suggesting that LAT1 inhibition reduced glucose utilization and glycolytic activities. As hexokinase 2 (HK2) expression remained unchanged (Suppl. Fig.S3C), the alteration in G-6-P levels might be attributed to LAT1-mediated changes in glucose uptake or downstream glycolytic pathways. To further assess the effect of LAT1 inhibition on glycolysis, we conducted a Seahorse assay in shLAT1 and scrambled control cells. As expected, LAT1 knockdown led to a marked reduction in glycolytic activity, as shown by decreased extracellular acidification rate (ECAR) (Fig. 3G). Notably, the knockdown of a non-TNBC cell line MCF-7 also reduced pyruvate, lactate, and G-6-P productions, suggesting that the findings may have broader implications beyond TNBC (Suppl. Fig.S3D, E).

### LAT1 enhances glycolytic activities via activating PKM2 and LDHA

One of the key enzymes in glycolysis is PKM2, which primarily catalyzes the conversion of phosphoenolpyruvate (PEP) to pyruvate [18]. To explore the mechanisms underlying LAT1's role in glycolysis, we conducted gene correlation analysis between LAT1 and glycolytic enzymes using the TCGA dataset. While no significant correlation was observed between LAT1 and HK2, and pyruvate kinase (PKLR) in breast cancer (Suppl. Fig.S4A, B), G6P Isomerase (GPI) and two isoforms of phosphofructose kinase 1 (PFK1) PFKL and PFKP were positively correlated with LAT1 (Suppl. Fig.S4C, D).

**Fig. 4:**
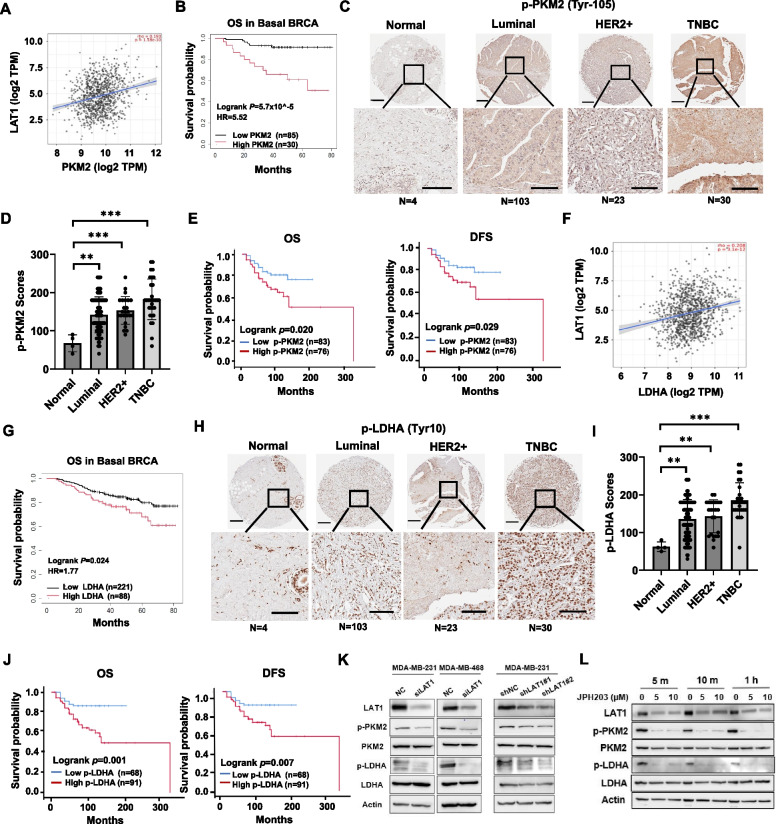
LAT1 enhances glycolytic activities via activating PKM2 and LDHA. **A** LAT1 and PKM2 expression levels in BC were retrieved from TCGA. The correlation between LAT1 and PKM2 expression was assessed using Spearman's rank correlation analysis. **B** Kaplan–Meier analysis for the OS in patients with high or low PKM2 expression levels in the basal BC subtype using TCGA data. **C-E** The levels of p-PKM2 (Tyr105) protein in normal and BC tissues were evaluated by IHC using a human breast cancer tissue microarray. An IHC staining score was calculated for p-PKM2. **C** Representative images are shown. Scale bar = 600 µm (Top); 300 µm (Inset). **D** Quantification of IHC staining for p-PKM2. **E** Kaplan–Meier plot of the OS and DFS in BC patients with high or low expression of p-PKM2 (Try105) based on tissue microarray IHC staining score. **F **LAT1 and LDHA expression levels in BC were retrieved from TCGA. The correlation between LAT1 and LDHA expression was assessed using Spearman's rank correlation analysis. **G** Kaplan–Meier plot of OS in the basal subtype of BC patients with high or low LDHA expression levels, using TCGA data. Survival curves were compared using the log-rank test.** H-J** Human breast cancer tissue microarrays were stained for p-LDHA (Tyr10) levels by IHC. **H** Representative images were shown. Scale bar = 600 µm (Top); 300 µm (Inset). **I** Quantification of IHC staining for p-LDHA. **J** Kaplan–Meier survival analysis of OS and DFS in BC patients with high or low p-LDHA (Tyr10) expression based on IHC staining scores. **K** The protein levels of LAT1, PKM2, p-PKM2, LDHA, and p-LDHA were measured by Western blotting in TNBC cells with transient or stable LAT1 knockdown. **L** MDA-MB231 cells were treated with JPH203 at different time points and doses as indicated. The protein levels of LAT1, PKM2, p-PKM2, LDHA, and p-LDHA were measured by immunoblotting. Three independent experiments were performed with a minimum of three biological replicates. ** *P* < 0.01; *** *P* < 0.001

Data retrieved from the TCGA dataset showed that the expression levels of G6P and three isoforms of PFK-1 were not associated with overall survival in breast cancer patients, suggesting these genes may have limited clinical relevance (Suppl. Fig.S4E, F). Notably, PKM2 expression was significantly related with LAT1 in breast cancer (Fig. 4A). In the basal subtype of breast cancer, patients with high PKM2 expression had significantly poor OS compared to those with low PKM2 expression (Fig. 4B). Furthermore, we performed IHC staining for p-PKM2 (Tyr-105) on a microarray containing 160 breast tissue cores. p-PKM2 expressions were elevated in Luminal, HER2 +, and TNBC tissues compared to normal breast tissues, with TNBC showing the highest expression levels (Fig. 4C, D). The survival analysis showed that patients with higher p-PKM2 expression had significantly shorter OS and DFS compared to those with lower p-PKM2 levels in all subtypes of breast cancer (Fig. 4E). Similarly, we examined the correlation between LDHA, a key enzyme in glycolysis responsible for converting pyruvate to lactate [19], and LAT1 by analyzing the TCGA data. A significant positive correlation between LDHA and LAT1 was identified (Fig. 4F). Survival analysis of TCGA data indicated that, in the basal subtype of breast cancer, patients with high LDHA expression had significantly shorter OS compared to those with lower LDHA expression (Fig. 4G). IHC staining of the breast cancer tissue arrays showed that p-LDHA (Tyr10) expression was elevated in Luminal, HER2 +, and TNBC tissues compared to normal breast tissues, with TNBC showing the highest levels of expression (Fig. 4H, I). The survival analysis indicated that patients with higher p-LDHA expressions had significantly shorter OS and DFS compared to those with lower p-LDHA levels (Fig. 4J). To further explore the relationship between LAT1 and the activities of PKM2 and LDHA, we conducted immunoblotting analyses in TNBC cells with silenced LAT1. Both transient and stable LAT1 knockdown in cells led to decreased expressions of p-PKM2 (Tyr-105) and p-LDHA (Tyr-10), suggesting that LAT1 enhances the activities of PKM2 and LDHA by promoting their phosphorylation (Fig. 4K). Notably, we observed that the inhibition of LAT1 did not significantly change the total protein levels of PKM2 and LDHA in MDA-MB-231 and 468 cells, which was not in line with the correlation findings using TCGA dataset. We reason that the discrepancy observed between tissues and cells is partly due to tumor heterogenous. The cancer tissues contain various subpopulations of cells while cell lines represent clonal populations and cannot capture the heterogeneity. Additionally, PKM2 and LDHA are highly expressed in MDA-MB-231 and MDA-MB-468 cells, making it difficult to detect moderate changes in their expression levels. Pharmacological inhibition of LAT1 with a LAT1 inhibitor JPH203 reduced the expressions of both p-PKM2 and p-LDHA, further suggesting that LAT1 acts as an upstream activator of PKM2 and LDHA in glycolysis (Fig. 4L). The mechanism of action of JPH203 is to bind to LAT1, thereby preventing the essential amino acids that LAT1 normally transport from entering the cell. Unexpectedly, we observed that JPH203 treatments reduced LAT1 protein levels in TNBC cells. Several studies showed that LAT1 inhibition by JPH203 treatment suppressed mTOR pathway as reflected as decreased phosphorylation levels of ribosomal protein S6K1 and eukaryotic translation initiation factor 4EBP1 [20, 21]. We speculate that the downregulation of mTOR/S6K1/4EBP1 pathway results in global or selective translational repression, possibly affecting LAT1 synthesis. Another possible explanation is the direct binding of JPH203 to LAT1, leading to the degradation of LAT1. It would be worthwhile to investigate this further in future studies. Overall, these data demonstrate that LAT1 promotes glycolysis by increasing the activities of PKM2 and LDHA.

### LAT1 promotes NAD^+^ de novo synthesis for glycolysis through L-Trp and QPRT

**Fig. 5:**
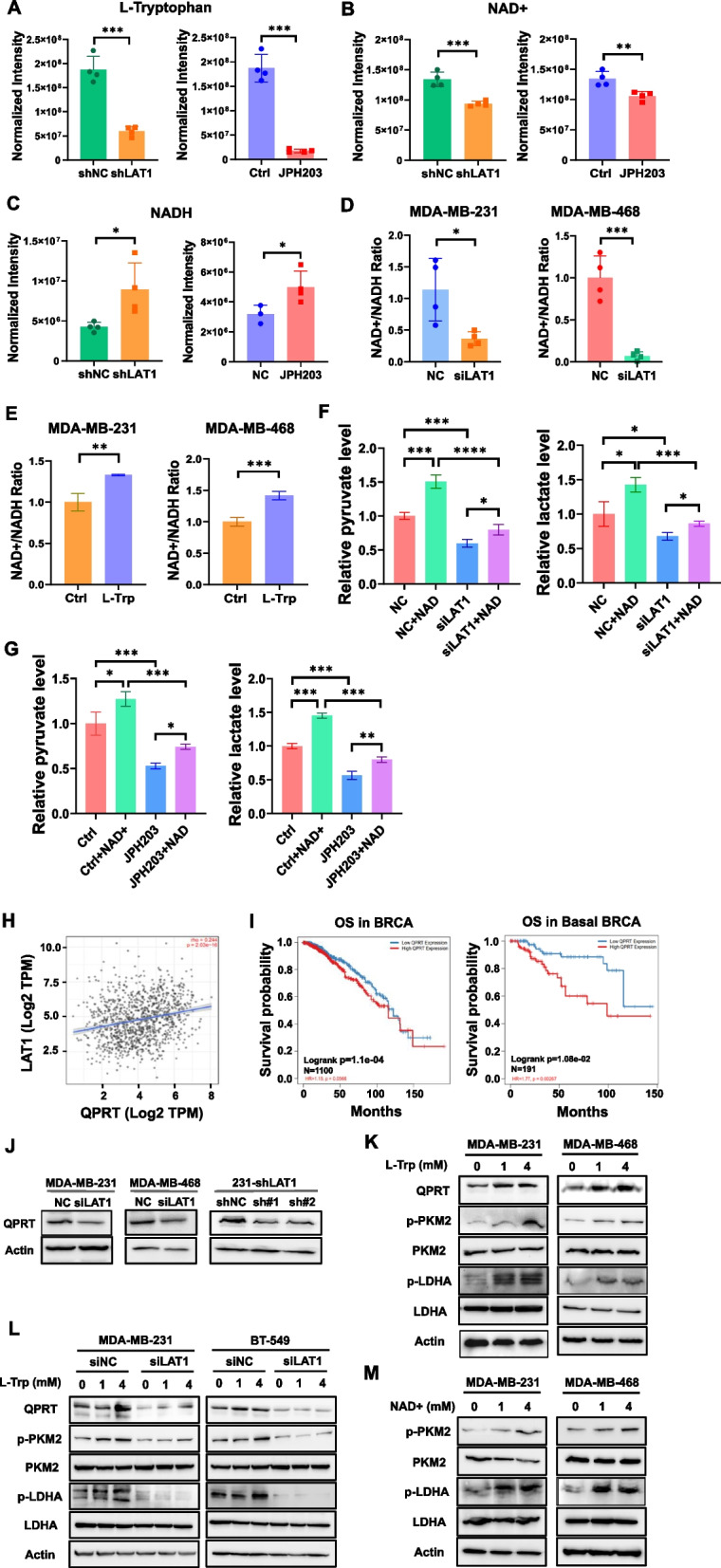
LAT1 promotes NAD + de novo synthesis through L-Trp/QPRT for glycolysis. **A-C** MDA-MB-231 cells were transfected with 100 nM LAT1 Smartpool siRNA or treated with 10 µM JPH203 for 10 min to inhibit the activity of LAT1. LC–MS-based metabolomics was performed to profile changes in cellular metabolites. The relative levels of intracellular (**A**) L-Tryptophan, **B** NAD^+^, and **C** NADH in LAT1-inhibited and control cells were measured by the metabolomics assay. Peak intensities were normalized for comparison (*n* = 4). **D** TNBC cells were transfected with 100 nM LAT1 Smartpool siRNA or control siRNA (NC) for 72 h. The relative cytosolic NAD^+^/NADH ratios were measured using a NAD^+^/NADH Quantification Kit.** E** TNBC cells were treated with 4 mM L-Tryptophan for 30 min. The relative cytosolic NAD +/NADH ratios were determined as described above. **F** MDA-MB-231 cells were transiently transfected with 100 nM Smartpool siLAT1 or a negative siRNA control (NC) for 72 h. The cells were then treated with either vehicle control or 4.0 mM NAD⁺ for 20 min. Pyruvate and lactate levels were measured using the corresponding assay kits. **G** MDA-MB-231 cells were treated with vehicle control, 4.0 mM NAD^+^, 10 µM JPH203, or a combination 4.0 mM NAD^+^ and 10 µM JPH203 for 10 min. Pyruvate and lactate levels were measured using the corresponding assay kits. **H** The correlation between LAT1 and QPRT in BC was evaluated using Spearman's rank correlation analysis of TCGA data. **I** QPRT expression and survival data were obtained from the TCGA database. Kaplan–Meier survival curves for OS were plotted for patients with high or low QPRT expression, both for overall BC patients (left) and for those with the basal subtype of BC (right). **J** LAT1 was silenced in TNBC cells using either siRNA transfection or lentiviral shRNA transduction. QPRT protein expression was assessed by immunoblotting. **K** MDA-MB-231 and MDA-MB-468 cells were incubated in amino acid–free medium supplemented with L-tryptophan for 30 min. The protein levels of QPRT, PKM2, p-PKM2, LDHA, and p-LDHA were evaluated by Western blotting. **L** MDA-MB-231 and BT549 cells were transiently transfected with 100 nM SmartPool siLAT1 or control siRNA (NC) for 72 h, followed by incubation in amino acid–free medium supplemented with L-tryptophan for 30 min. The protein levels of QPRT, PKM2, p-PKM2, LDHA, and p-LDHA were analyzed by immunoblotting. **M** Cells were treated with NAD^+^ for 20 min. The protein levels of PKM2, p-PKM2, LDHA, and p-LDHA were measured by immunoblotting. * *P* < 0.05; ** *P* < 0.01; *** *P* < 0.001. Three independent experiments were performed with a minimum of three biological replicates

L-Tryptophan is one of major substrates of LAT1 in cancer cells. As expected, the uptake of L-Tryptophan as well as L-Leucine significantly decreased in MDA-MB-231 cells expressing shLAT1 or treated with JPH203 (Fig. 5A, Suppl. Fig.S5A). Notably, we also observed a significant reduction in cellular NAD^+^ levels and an increase in NADH levels (Fig. 5B, C) following LAT1 inhibition in MDA-MB-231 cells, which is consistent with the results of the pathway enrichment analysis. To confirm these findings, we measured the cytosolic NAD^+^/NADH ratio in MDA-MB-231 and MDA-MB-468 cells transfected with siLAT1 or scrambled controls. LAT1 silencing resulted in a more than 60% reduction in the NAD^+^/NADH ratio in both TNBC cell lines (Fig. 5D). Since L-Tryptophan serves as a precursor for the de novo synthesis of NAD^+^ as well as a substrate for LAT1, we speculated whether LAT1 increases the NAD^+^/NADH ratio by facilitating the transport of L-Tryptophan into the cells. Indeed, we observed a significant increase in the NAD^+^/NADH ratio in TNBC cells following L-Tryptophan treatment (Fig. 5E). We then investigated whether NAD^+^ contributes to LAT1-mediated regulation of glycolysis by supplementing LAT1 knockdown cells with NAD^+^ and measuring the intracellular pyruvate and lactate levels. We showed that silencing LAT1 via siRNA transfection led to decreased pyruvate/lactate levels, while supplementation with NAD^+^ partially restored the reduced pyruvate/lactate levels in siLAT1-transfected MDA-MB-231 and BT-549 cells compared to cells treated with vehicle controls (Fig. 5F, Suppl.Fig.S5B). The similar results were obtained in cells treated with JPH203 (Fig. 5G). The de novo synthesis pathway of NAD + is driven by the rate-limiting enzyme quinolinate phosphoribosyltransferase (QPRT), which is overexpressed in various cancer types, including breast cancer (Suppl. Fig.S5C). Utilizing the TCGA dataset, we found LAT1 expressions are positively correlated with QPRT levels in breast cancer (Fig. 5H). Patients with high QPRT expressions exhibited significantly shorter OS compared to those with low QPRT levels in overall breast cancer and in the basal-like TNBC subtype (Fig. 5I). Additionally, QPRT expression was significantly reduced following LAT1 inhibition by siRNA or shRNA in TNBC cells, suggesting that LAT1 may directly or indirectly regulate QPRT expression (Fig. 5J). Treatment of TNBC cells with L-Tryptophan upregulated QPRT levels, as well as p-PKM2 and p-LDHA (Fig. 5K). Silencing LAT1 in TNBC cells via siRNA transfection abolished the L-Tryptophan-induced upregulation of QPRT, p-PKM2, and p-LDHA (Fig. 5L), suggesting an essential role of LAT1 for L-Tryptophan-mediated regulation of QPRT and glycolysis. To further investigate whether increased NAD^+^ concentrations could enhance glycolysis by activating PKM2 and LDHA, we treated TNBC cells with NAD^+^ and observed that both p-PKM2 and p-LDHA levels were induced in MDA-MB-231 and MDA-MB-468 cells upon NAD^+^ treatment (Fig. 5M). Collectively, LAT1 promotes glycolysis by increasing both cellular uptake of L-Tryptophan and the QPRT expression.

### QPRT mediates LAT1-induced upregulation of p-PKM2 and p-LDHA

**Fig. 6:**
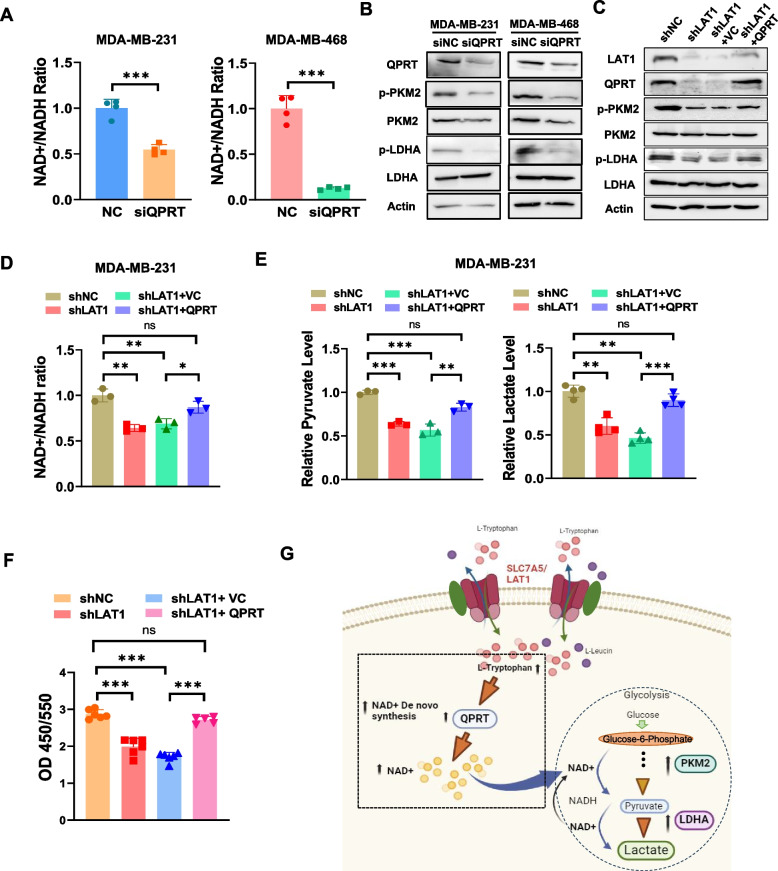
QPRT mediated LAT1-induced upregulation of p-PKM2 and p-LDHA. **A-B** MDA-MB-231 and MDA-MB-468 cells were transfected with 100 nM SmartPool QPRT siRNA or a negative control siRNA (NC) for 72 h. **A** The relative cytosolic NAD +/NADH ratios were measured using a NAD/NADH Quantification Kit. **B** The protein levels of QPRT, PKM2, p-PKM2, LDHA, and p-LDHA were measured by Western blotting. **C-F** The shLAT1-expressing MDA-MB-231 cells were transfected with a QPRT-expressing plasmid or a vector control. **C** The protein levels of LAT1, QPRT, PKM2, p-PKM2, LDHA, and p-LDHA were measured by immunoblotting.** D** Relative cytosolic NAD +/NADH ratios were measured. **E** Pyruvate and lactate productions were assessed using pyruvate and lactate assay kits, respectively. **F** Cell proliferation assay was performed using BrdU assay kits. **G** A graphic summary of the proposed LAT1/QPRT/PKM2/LDHA signaling pathway. Three independent experiments were performed with a minimum of three biological replicates. * *P* < 0.05; ** *P* < 0.01; *** *P* < 0.001

We then examined the role of QPRT in LAT1-mediated regulation of glycolysis. Silencing QPRT in TNBC cells via siRNA transfection resulted in a significant reduction in the NAD^+^/NADH ratio compared to cells transfected with scrambled controls (Fig. 6A). The expression of p-PKM2 and p-LDHA was downregulated in QPRT knockdown TNBC cells, indicating that QPRT promotes the activities of these two glycolytic enzymes (Fig. 6B). To determine whether LAT1 regulates glycolysis through QPRT, we overexpressed QPRT in LAT1 knockdown MDA-MB-231 cells by transfecting a plasmid containing a QPRT-encoding cassette (Fig. 6C). The overexpression of QPRT resulted in increased levels of LAT1, p-PKM2 and p-LDHA in LAT1-silenced cells (Fig. 6C). We then examined the impact of QPRT restoration on the inhibition of glycolysis induced by LAT1 silencing. Consistent with our previous findings, LAT1 inhibition by shRNA caused a significant decrease in the NAD^+^/NADH ratio, pyruvate, and lactate levels in MDA-MB-231 cells, while shLAT1-expressing cells transfected with the QPRT plasmid showed a significant increase in the NAD^+^/NADH ratio, pyruvate, and lactate levels compared to those transfected with a control vector (Fig. 6D, E). Additionally, restoration of QPRT expression rescued the impaired cell proliferation and migration caused by LAT1 silencing (Fig. 6F, Suppl. Fig.S6). Together, these results demonstrate that QPRT mediates LAT1-regulated glycolysis. A graphic summary of a proposed LAT1/QPRT/PKM2/LDHA signaling pathway is presented in Fig. 6G.

### Knockdown of LAT1 sensitizes resistant TNBC cells to Doxorubicin

**Fig. 7:**
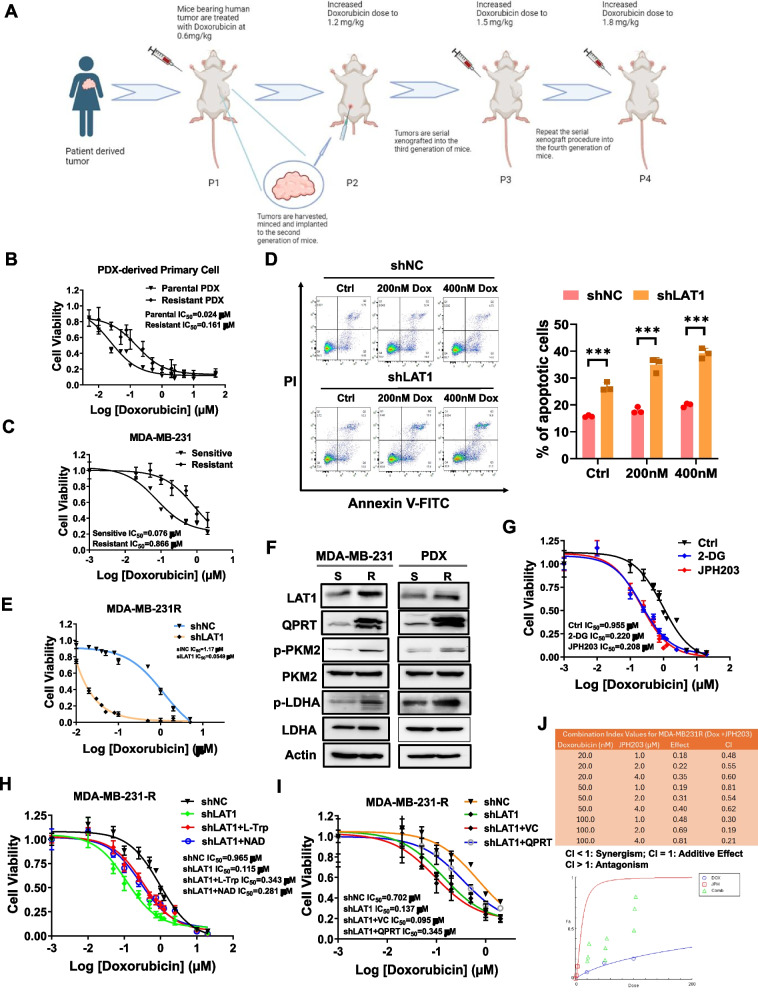
Knockdown of LAT1 sensitized resistant TNBC cells to Doxorubicin. **A** A schematic illustration of the establishment of an acquired Dox-resistant PDXmouse model. The PDX tumor without Dox treatment was set up as a control. After four passages, both the parental and resistant PDX tumors were harvested, and primary cells were dissociated using enzymatic digestion. **B** The dissociated primary cells were seeded onto a 96-well plate and treated with different concentrations of Dox for 72 h. The IC_50_ values of Dox in the cells were determined using an MTT assay. **C** Parental and Dox-resistant MDA-MB-231 cells were treated with different concentrations of Dox for 72 h. The IC_50_ values were determined using an MTT assay. **D** The MDA-MB-231-R cells were transfected with 100 nM siLAT1 or siNC for 24 h. The cells were then treated with vehicle control, 200 nM, or 400 nM Dox for 48 h followed by apoptosis assay. **E** MDA-MB-231-R stably LAT1 knockdown cells (shLAT1) and control cells (shNC) were treated with Dox at different doses. The cell viability was measured using the colorimetric BrdU incorporation assay. **F** The protein levels of LAT1, QPRT, PKM2, p-PKM2, LDHA, and p-LDHA were measured by immunoblotting in sensitive and Dox-resistant MDA-MB-231 cells, as well as in primary cells dissociated from parental and Dox-resistant PDX tumors. **G** MDA-MB-231-R cells were treated with a vehicle control, 10 mM 2-DG, or 5 µM JPH203 in combination with varying concentrations of Dox for 72 h. IC₅₀ values were determined using an MTT assay. **H** shLAT1-expressing MDA-MB-231-R cells were treated with vehicle control, 1 mM L-Trp, or 1 mM NAD⁺, along with varying concentrations of Dox for 72 h. The IC₅₀ values of Dox in the cells were then determined using the MTT assay. **I** The shLAT1-expressing MDA-MB-231 cells were transfected with either a QPRT-expressing plasmid or a vector control, followed by incubation with varying concentrations of Dox for 72 h. The IC₅₀ values of Dox in the cells were then determined using the MTT assay. **J** MDA-MB-231-R cells were treated with Dox alone, JPH203 alone, or a combination of both drugs at the indicated doses for 72 h. Cell viability was assessed using MTT assays, and combination index (CI) analysis was performed. The figure illustrating the combined effects was generated using CompuSyn software. Three independent experiments were performed with a minimum of three biological replicates.* *P* < 0.05; *** *P* < 0.001

Chemotherapy resistance in TNBC patients has been a long-standing challenge which leads to higher rates of distant metastases, and poorer overall survival compared to other breast cancer subtypes. To investigate whether LAT1 elevation contributes to chemo-resistance in TNBC, we established an induced Dox-resistant in vivo PDX mouse model by treating mice implanted with human TNBC tumors with increasing doses of Doxorubicin over eight weeks, across four tumor passages. Primary cells were then isolated from both vehicle control tumors (PDX-Parental) and Dox-treated tumors (PDX-Resistant) through enzymatic digestion. A schematic illustration of this process is shown in Fig. 7A. The IC_50_ values of Doxorubicin in the tumor-dissociated primary cells were determined using the MTT assay. The PDX-Resistant cells exhibited approximately seven-fold higher IC_50_ value compared to the PDX-Parental cells (Fig. 7B). We also generated a Dox-resistant MDA-MB-231 cell (MDA-MB-231-R) by continuously treating the cells with gradually increasing doses of Doxorubicin over months in vitro. The MDA-MB-231-R cells exhibited an 11-fold increase in the IC_50_ value of Doxorubicin compared to the parental MDA-MB-231 cells (Fig. 7C). We then established a stable LAT1 knockdown in MDA-MB-231R cells by transducing with lentivirus expressing shNC or shLAT1, followed by puromycin selection (Suppl. Fig.S7A). We then accessed the effect of LAT1 knockdown on the sensitivity of MDA-MB-231-R cells to Doxorubicin by an apoptosis assay. The results showed that LAT1 knockdown alone induced apoptosis, and Doxorubicin treatment significantly increased cell death in the LAT1-knockdown cells compared with the control cells, indicating that LAT1 silencing enhanced cytotoxicity of Doxorubicin in the resistant cells (Fig. 7D). In addition, we assessed cell viability using the MTT assay. Consistent with the cell apoptosis data, LAT1 knockdown significantly reduced the IC50 of Doxorubicin in MDA-MB-231-R cells, with the IC50 decreasing from 1.17 µM in control cells to 0.0549 μM in LAT1 knockdown cells (Fig. 7E). Next, we determined the expression levels of glycolytic enzymes by immunoblotting in parental and Dox-resistant MDA-MB-231 cells, as well as in PDX-dissociated primary cells. We showed the expression levels of LAT1, QPRT, p-PKM2, and p-LDHA were higher in Dox-resistant cells relative to their respective controls (Fig. 7F), indicating an increased glycolytic activity in Dox-resistant cells. Inhibition of glycolysis by 2-DG enhanced the sensitivity of resistant cells to Doxorubicin, which was similar to the effect observed with JPH203 (Fig. 7G). Furthermore, the supplementation of L-Trp or NAD + partially reversed the enhanced sensitivity to Doxorubicin induced by LAT1 knockdown (Fig. 7H). We also investigated the role of QPRT in LAT1 silencing-induced sensitivity to Dox in resistant TNBC cells. Stable downregulation of LAT1 by shRNA significantly decreased the IC_50_ value in MDA-MB-231-R cells. However, LAT1 knockdown cells transfected with QPRT plasmids upregulated IC_50_ value compared to cells transfected with a control vector, suggesting that overexpression of QPRT in LAT1-silenced cells partially reverses the enhanced sensitivity to Dox (Fig. 7I). In addition, the combination of Doxorubicin and LAT1 inhibitor JPH203 induced higher cytotoxicity in resistant MDA-MB-231-R cells, as well as in Dox-sensitive MDA-MB-468 cells (Fig. 7J, Suppl. Fig.S7B). The combination index (CI) analyzed by CompuSyn software showed the strong synergism between Doxorubicin and JPH203 [22]. Overall, our data demonstrates that LAT1 inhibition greatly improves the efficacy of Doxorubicin in TNBC cells.

## Discussion

Triple-negative breast cancer is characterized by a high grade, a high proposition index, and tumor necrosis, making it especially dependent on glucose metabolism [23]. Accumulating studies indicate that the Warburg effect mediates TNBC cell proliferation, metastasis, recurrence, immune escape, and multidrug resistance [24]. High expressions of glucose transporters, lactate transporters and glycolysis-related enzymes have been observed in TNBC [23]. In addition, metabolic rewiring through dysregulated amino acid uptake such as glutamine has been identified as a significant factor to enhance glycolysis in TNBC progression and chemotherapy resistance by fueling key metabolic processes [25, 26]. In this study, we have identified that overexpression of LAT1, a transporter of several essential amino acids, contributes to the elevated glycolytic activity in TNBC. Our GSEA analysis using TCGA data revealed that patients with higher LAT1 expression have a more enriched glycolysis pathway compared to oxidative phosphorylation. Knockdown of LAT1 in TNBC cells significantly reduced glycolysis as measured by lactate production and ECAR in real time. LAT1 (SLC7A5) belonged to the solute carrier family 7 (SLC7), is responsible for the transport of eight essential amino acids including leucine, threonine, methionine, and tryptophan, as well as some hormones and drugs [27]. Although LAT1 expression has been strongly associated with poor prognosis in breast cancer, the underlying molecular mechanisms are less studied. It has been reported that L-leucine, one of the major LAT1 substrates, stimulates the mechanistic target of rapamycin kinase complex 1 (mTORC1), known as a central hub of nutrient signaling [28–32]. LAT1 enhanced breast cancer cells proliferation, invasion, and migration through mTORC1 pathway, suggesting a connection with LAT1-mediated uptake of leucine [33]. In this study, we reveal a novel mechanism in which LAT1 can indirectly but significantly affect key glycolytic enzyme activities through its major substrate L-Tryptophan. We showed that LAT1 inhibition reduced the uptake of L-Tryptophan in TNBC cells, and the addition of L-Tryptophan enhanced the activities of PKM2 and LDHA.

L-Tryptophan serves as a precursor for the de novo NAD^+^ synthesis in the kynurenine pathway [34]. Not surprisingly, the L-Tryptophan treatment increased the ratio of NAD +/NADH in TNBC cells. Nicotinamide adenine dinucleotide (NAD^+^) is an essential coenzyme for various physiological processes including energy metabolism, mitochondrial functions, DNA repair, and epigenetic modifications [35, 36]. Many of these NAD^+^ -dependent signaling pathways are dysregulated in cancer, and the NAD +/NADH redox ratio is five times higher in cancer cells compared to the normal cell population [37]. Therefore, targeting NAD^+^ synthesis has been considered as a new therapeutic concept for cancer treatment. NAD^+^ is required as coenzyme for two glycolytic enzymes GAPDH and LDHA; therefore, an increased NAD^+^/NADH ratio favors glycolysis [36]. Our enrichment pathway analysis showed nicotinate and nicotinamide metabolism as one of the top enriched pathways in LAT1 expressing cells. Knockdown of LAT1 significantly reduced cellular level of NAD^+^, and exogenous NAD^+^ treatment rescued the pyruvate/lactate production in LAT1 knockdown cells, suggesting that LAT1 promotes glycolysis partially through NAD^+^- dependent pathways. Quinolinate phosphoribosyltransferase (QPRT) is a rate-limiting enzyme in the kynurenine pathway for NAD^+^ synthesis through de novo pathway. Interestingly, QPRT has been reported to be highly expressed in breast cancer and is associated with high tumor aggressiveness and poor patient outcomes [38, 39]. Our data showed that knockdown of LAT1 decreased the expression level of QPRT, and overexpression of QPRT increased the pyruvate and lactate production, NAD^+^/NADH ratio, and cell proliferation in LAT1 knockdown cells, suggesting that LAT1 promotes NAD^+^ production through both L-Tryptophan uptake and NAD^+^ synthesis enzymes. The increased NAD^+^ subsequently drives the activities of PKM2 and LDHA for glycolysis. It is not clear how LAT1 enhances QPRT activities. We reason that the excessive import of L-Tryptophan due to high expression of LAT1 may cause positive feedback in cells to activate QPRT for NAD^+^ synthesis. In addition to pyruvate and lactate, our data showed that inhibition of LAT1 reduced the level of G-6-P while the protein level of phosphor- and total hexokinase 2 (HK2) remained unchanged. A study reported that NAD + deletion due to inhibition of NAMPT, an enzyme essential for NAD + biosynthesis leads to the reduction of glycolytic intermediates including G-6-P [40]. We speculate that decreased NAD⁺ by LAT1 knockdown can indirectly reduce G-6-P by slowing glycolysis, causing feedback inhibition on glucose uptake. Further investigation is warranted to elucidate the precise mechanisms through which LAT regulates QPRT activity and G-6-P production.

Unlike glucose transporters, LAT1 is regarded as a cancer-specific amino acid transporter with its protein expression predominantly localized to the plasma membrane of cancer cells, which makes LAT1 a promising molecular target for cancer treatment [13]. JPH203, a highly selective LAT1 inhibitor, has been developed and tested across multiple cancers with high efficacy in killing cancer cells [41–45]. JPH203 has completed phase I (UMIN000016546) and phase II (UMIN000034080) clinical trials against biliary tract cancer, showing promising results in terms of safety and efficacy [46, 47]. Consistent with these observations, we showed JPH203 inhibited TNBC tumor growth both in vitro and in vivo, and synergically improved the efficacy of chemotherapy in TNBC resistant cells. Taken together, our study uncovers a novel mechanism in which LAT1 drives TNBC progression and drug resistance by enhancing glycolysis activities through L-Trp/NAD^+^/QPRT/LDHA pathway. Notably, although this investigation focused on TNBC models due to the lack of targeted therapies for this subtype, we showed the proposed mechanism was also relevant to other molecular subtypes of breast cancer, suggesting that the findings may have broader implications beyond TNBC.

## Conclusions

Our findings identify a novel role of LAT1 in promoting TNBC progression and chemo-resistance by amplifying the Warburg effect. Elucidating LAT1-dependent signals in cancer metabolic rewiring highlights the potential of LAT1-targeted therapies for clinical applications.

## Supplementary Information

Supplementary Material 1.

## Data Availability

No datasets were generated or analysed during the current study.

## Abbreviations

TNBC: Triple Negative Breast Cancer
LAT1: L-Type amino acid transporter 1
QPRT: Quinolinate phosphoribosyltransferase
PKM2: Pyruvate kinase M2
LDHA: Lactate dehydrogenase A
Dox: Doxorubicin
PDX: Patient-derived xenograft
BrdU: 5-Bromo-2'-deoxyuridine
IF: Immunofluroscent staining
OE: Overexpression
VC: Vector control
NC: Negative control
NAD: Nicotinamide Adenine Dinucleotide
NADH: Nicotinamide Adenine Dinucleotide (Reduced Form)
L-Trp: L-Tryptophan
LCMS: Liquid Chromatography-Mass Spectrometry
