# Correction: Amino acid transporter LAT1 (SLC7A5) promotes metabolic rewiring in TNBC progression through the L-Trp/QPRT/NAD^+^ pathway

**DOI:** 10.1186/s13046-025-03611-4

**Published:** 2025-12-12

**Authors:** Margot Y. Fedoroff, Lei Zhao, Shaomin Wang, Leili Saeednejad Zanjani, Alok Bhushan, Haifeng Yang, Karen M. Bussard, Stephen C. Peiper, Jun He

**Affiliations:** 1 Department of Pathology and Genomic Medicine, Sidney Kimmel Cancer Center, Thomas Jefferson University, Philadelphia, PA USA; 2 Department of Pharmaceutical Sciences, Jefferson College of Pharmacy, Thomas Jefferson University, Philadelphia, PA USA; 3 Department of Pharmacology, Physiology & Cancer Biology, Sidney Kimmel Cancer Center, Thomas Jefferson University, Philadelphia, PA USA

**Correction: J Exp Clin Cancer Res 44**,** 190 (2025)**

**https://doi.org/10.1186/s13046-025-03446-z**

Following publication of the original article [1], the authors found that Dr. Leili Saeednejad Zanjani was not included in the final author list despite her contributions. The details are provided below:

**Current authors**: Margot Y. Fedoroff1†, Lei Zhao1†, Shaomin Wang1, Alok Bhushan2, Haifeng Yang1, Karen M. Bussard3, Stephen C. Peiper1 and Jun He1*.

**Updated author list**: Margot Y. Fedoroff1†, Lei Zhao1†, Shaomin Wang^1^, Leili Saeednejad Zanjani^1^, Alok Bhushan^2^, Haifeng Yang^1^, Karen M Bussard^3^, Stephen C Peiper^1^, Jun He^1^*.

The correction does not compromise the validity of the conclusions and the overall content of the article. The author group has been updated above and the original article [1] has been corrected.
