# Supplementary material for: Amino acid transporter LAT1 (SLC7A5) promotes metabolic rewiring in TNBC progression through the L-Trp/QPRT/NAD+ pathway
Source: J Exp Clin Cancer Res. 2025 Jul 3;44:190. doi: 10.1186/s13046-025-03446-z (PMC12224598; doi:10.1186/s13046-025-03446-z)
Supplement: Supplementary file 1 — Supplementary Material 1. [file 13046_2025_3446_MOESM1_ESM.docx]

**Supplemental Materials**

**Material and Methods**

Wound Healing Assay

BT-549 cells were transiently transfected with 100 nM Smartpool LAT1 siRNA or a negative siRNA control (NC) for 72 h. A wound healing assay was then performed to assess cell migration ability. Cells were seeded in a 6-well plate at a density of 2 × 10⁵ cells per well and cultured until reaching approximately 80–90% confluence. A sterile 1 mL pipette tip was used to create a linear scratch across the monolayer, simulating a wound. Detached cells and debris were gently removed by washing with phosphate-buffered saline (PBS). Fresh medium was then added. Images of the wound area were captured at 0 hours and 8h using a phase-contrast microscope. The wound closure was quantified by measuring the remaining gap area using ImageJ software, and data were presented as a percentage of wound closure relative to the initial scratch width. Each experiment was performed in triplicate to ensure reproducibility.

**Supplementary Figures and Figure legends**

**Fig.S1**

**A**

**
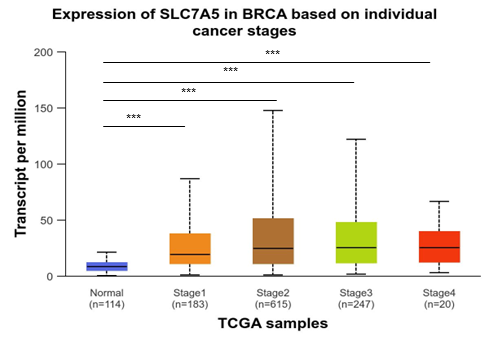
**

**B**

**
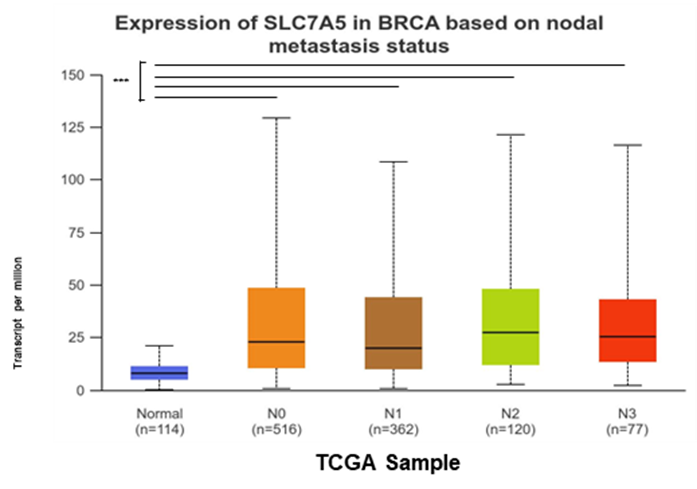
**

**Suppl Fig. S1**. LAT1 mRNA levels and clinical data for breast cancer (BC) were retrieved from the TCGA database. (**A**) LAT1 expression levels in normal breast tissues and in breast cancer (BC) at different stages (1-4) were compared. LAT1 expression was significantly higher in BC tissues at all stages compared to non-cancerous tissues, but no significant difference was observed across the cancer stages. (**B**) LAT1 levels in breast cancer (BC) with different nodal metastasis statuses (N0–3) were compared to normal breast tissues. LAT1 expression was significantly higher in BC tissues than in normal breast tissues. However, no significant difference was observed between BC with or without nodal metastasis.

**Fig.S2**

**A**


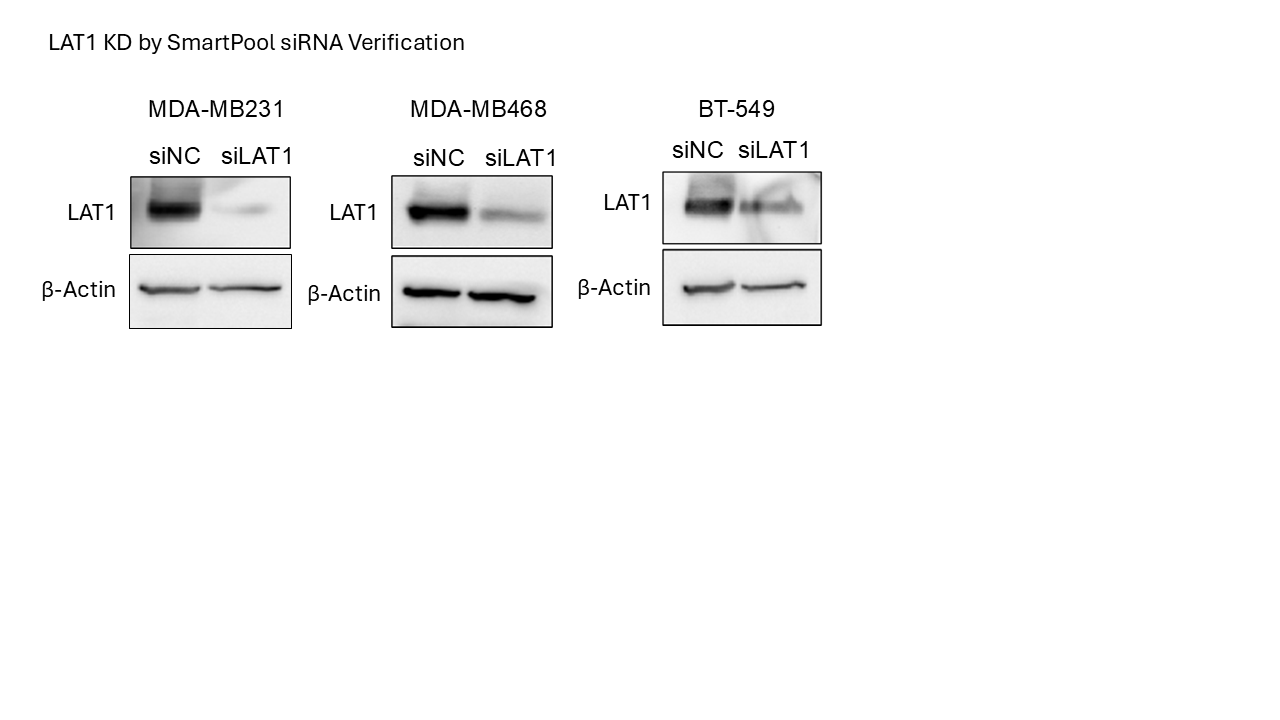


**B**


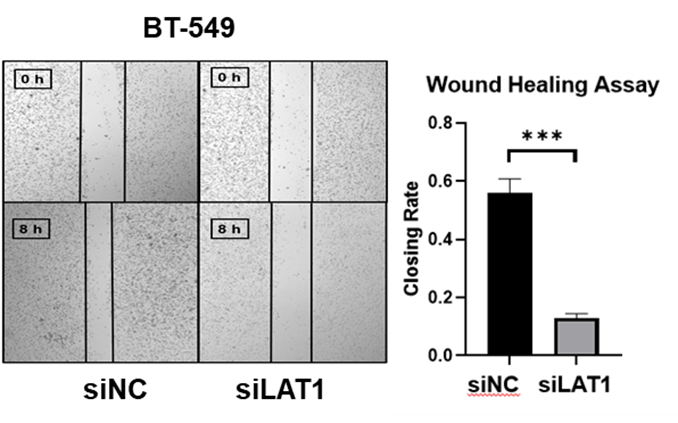


**C**

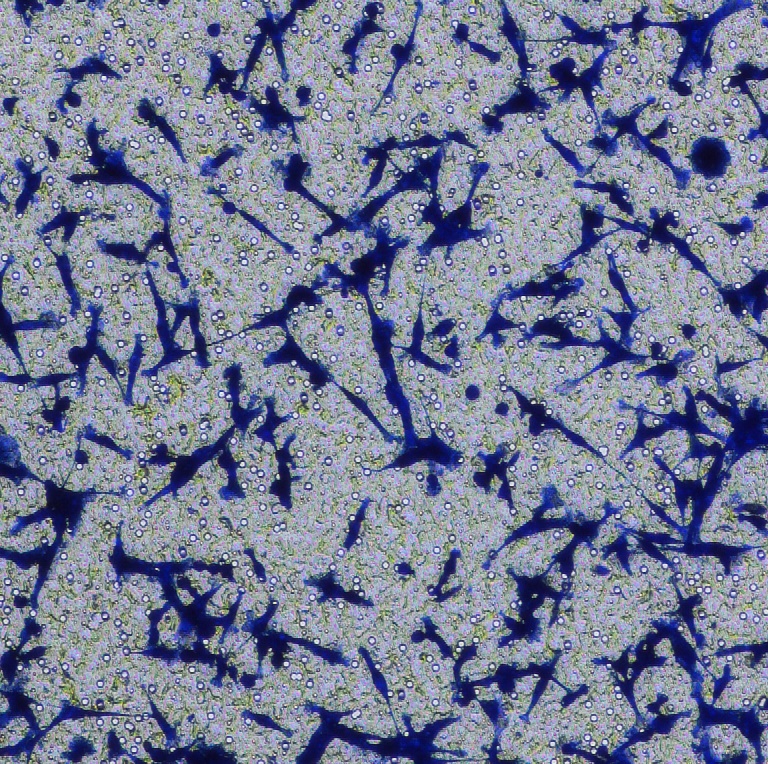

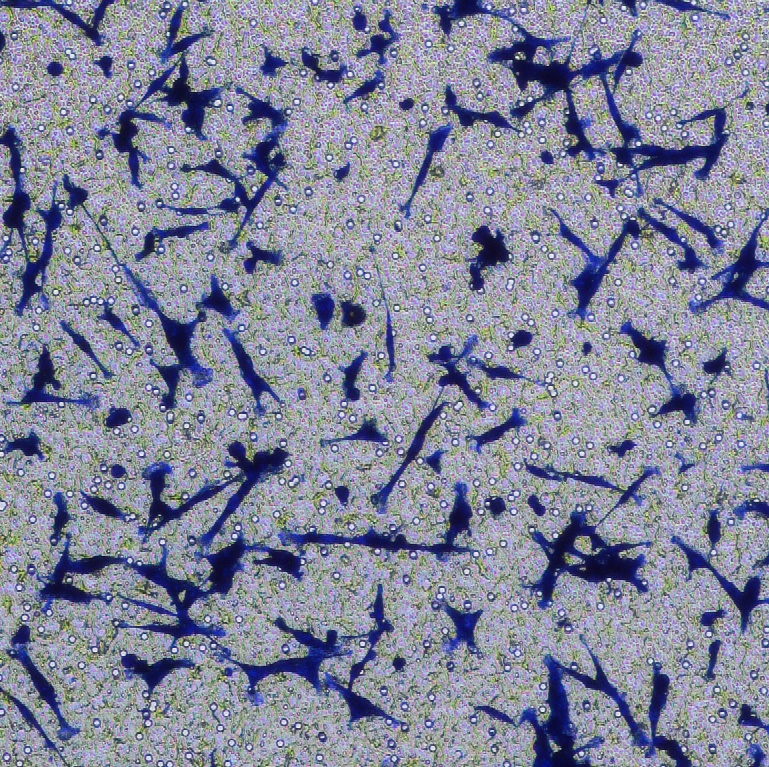


**siNC**

**siLAT1**

**Mitomycin C**

**D**


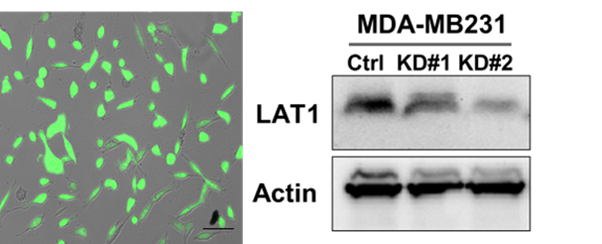


**E**


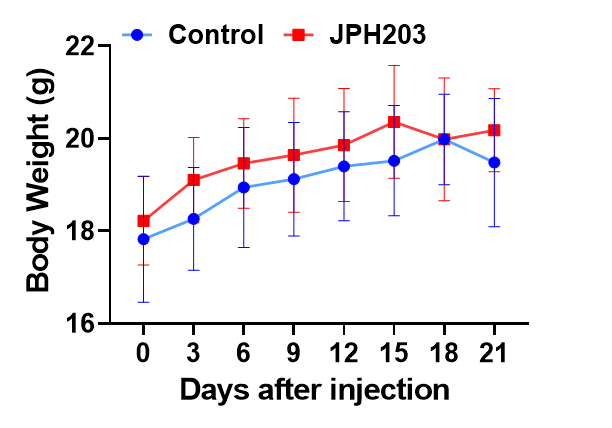


**Suppl Fig. S2**. **(A)** Cells were transiently transfected with 100 nM Smartpool LAT1 siRNA or a negative siRNA control (NC) for 72 h. LAT1 expression was measured using immunoblotting assay. (**B**) BT-549 cells were transfected with 100 nM siNC or siLAT1 for 72 h. A wound healing assay was then performed to assess cell migration ability. The gaps at 0 h and 8 h were imaged and quantified. Left: Representative images. Right: Quantification of the closing rate of the gaps. (**C**) MDA-MB-231 cells were transfected with 100 nM siNC or siLAT1 for 72 h, followed by treatment with either PBS (control) or 10 µg/mL mitomycin C (MMC) for 3 h. The cells were then harvested and subjected to a transwell migration assay. Left: representative images. Right: quantification of migrated cells. (**D**) MDA-MB-231 cells were transduced with lentivirus expressing GFP-tagged shNC or shLAT1, followed by puromycin selection. After selection, cells were sorted by flow cytometry to isolate the GFP-positive population. Left: GFP fluorescence in sorted cells. Right: Knockdown efficiency was confirmed by Western blot analysis. (**E**) Body weights of female C57BL/6 mice injected with PY8119 TNBC cells were measured every three days (n = 5 per group). *** P ˂ 0.001.

**Fig. S3**

**A**


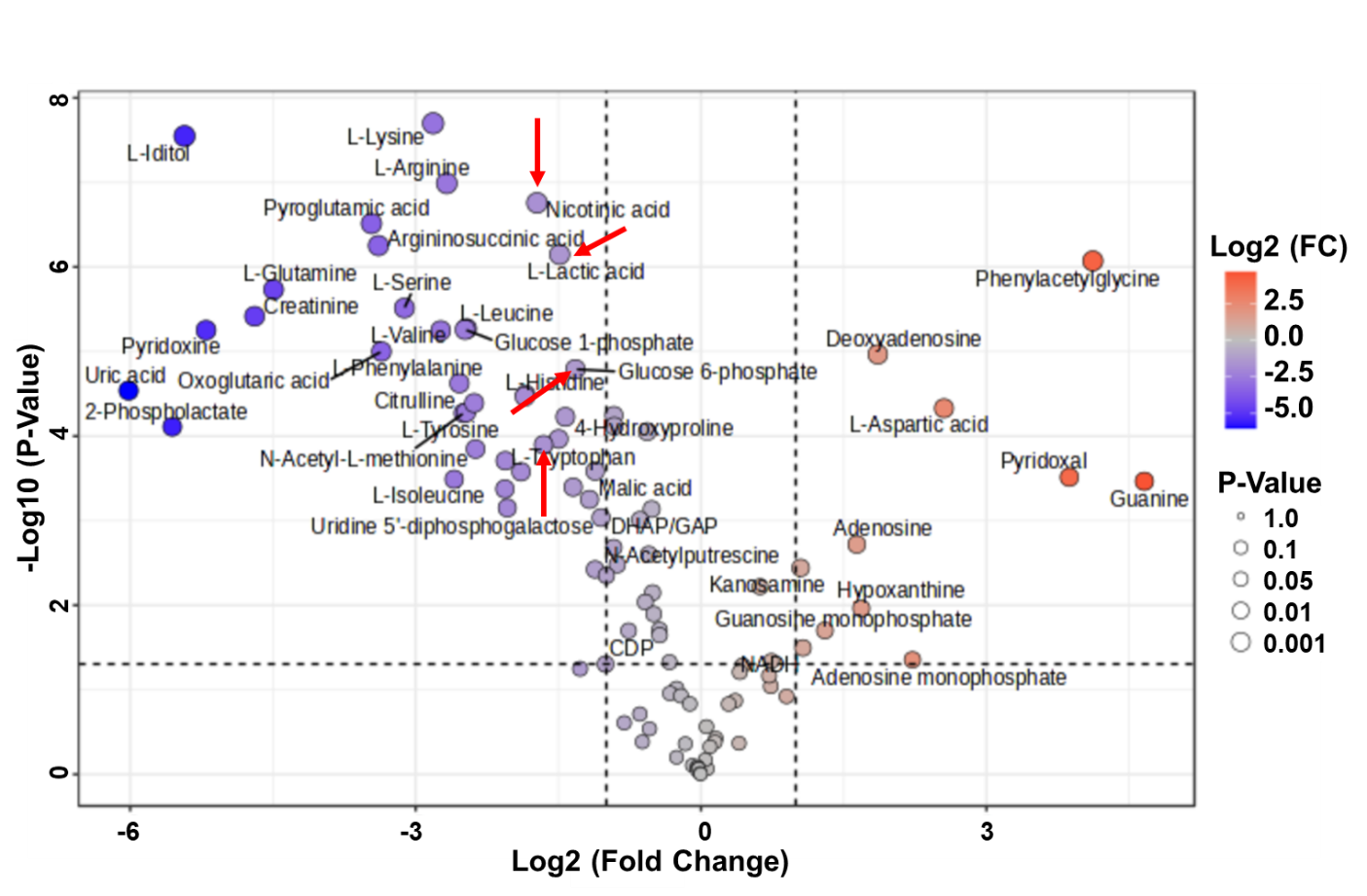


**B**

**
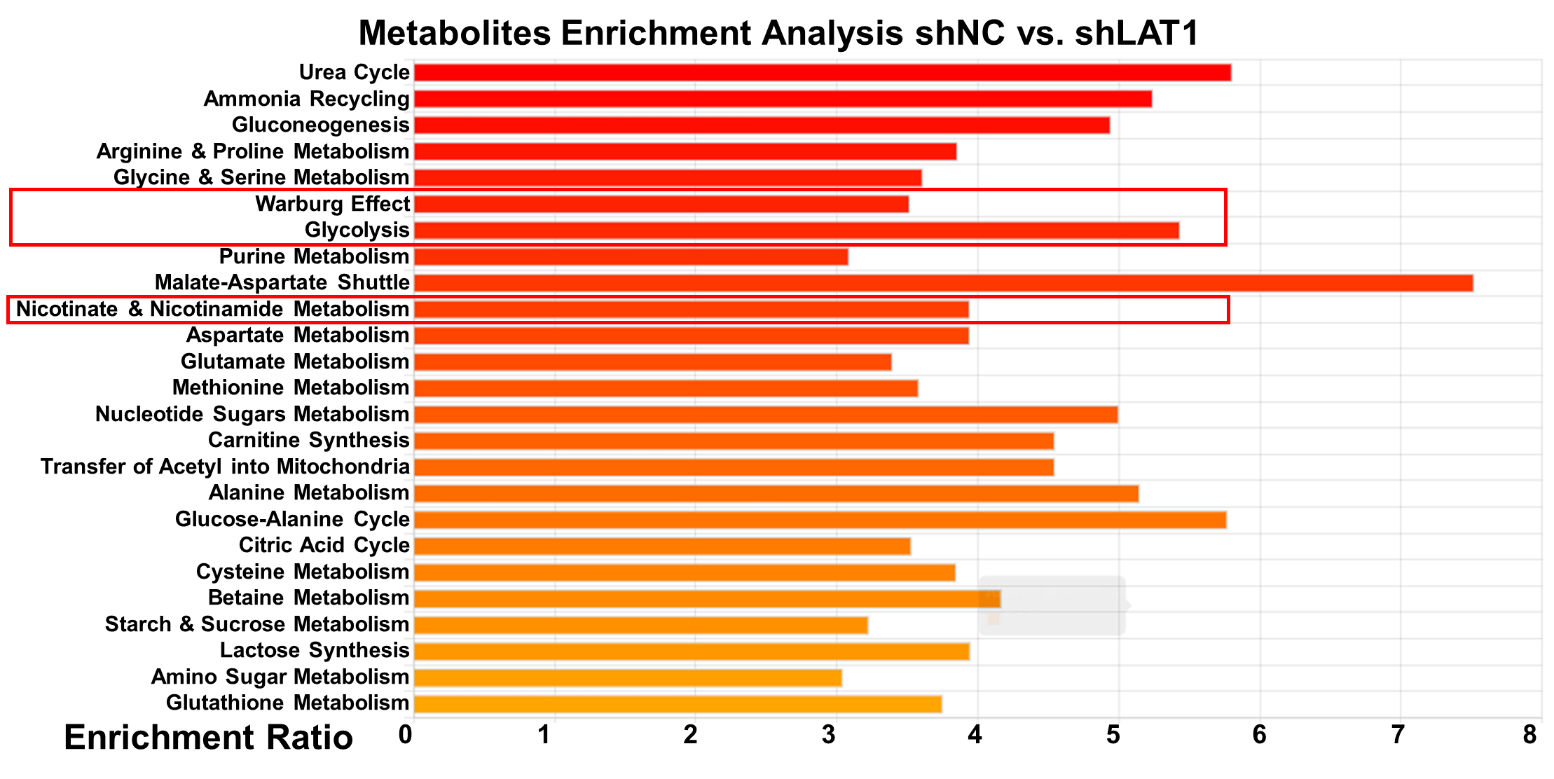
**

**C D**


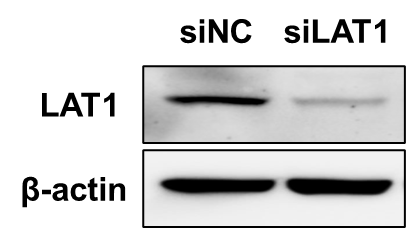

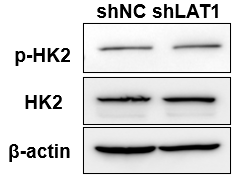


**E**

**
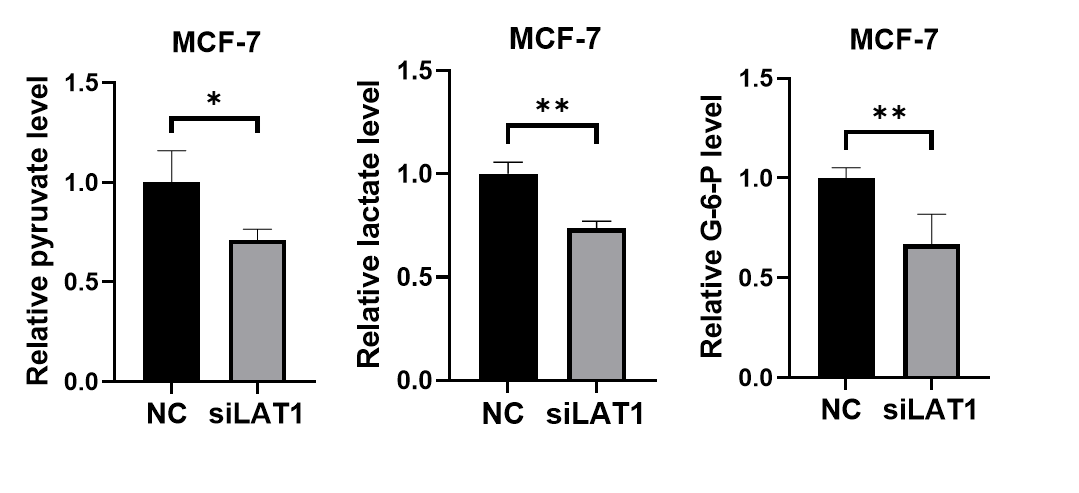
**

**Suppl Fig. S3**. (**A**) Volcano plot of the top 50 most significantly altered metabolites identified by LC-MS. The volcano plot shows the fold-change (x-axis) versus the significance (y-axis) of the identified metabolites in LAT1 shRNA and NC transfected MDA-MB231 cells. The significance (normalized p-value) and the fold-change are converted to –Log10 (p-value) and Log2 (fold-change), respectively. The vertical and horizontal dotted lines show the cut-off of fold change = ±1, and of p-value = 0.05, respectively. The volcano plot was generated using MetaboAnalyst. Arrows indicate the significantly changed intermediate metabolites of the glycolytic pathway. (**B**) Metabolite enrichment analysis was performed on control and shLAT1-expressing MDA-MB-231 cells to analyze the differentially expressed metabolites using MetaboAnalyst software. The bar chart shows the enriched metabolic pathways of the differential metabolites. (**C**) p-HK2 and total HK2 protein levels in shNC- and shLAT1-expressing MDA-MB-231 cells were measured by immunoblotting. (**D**) MCF7 cells were transfected with 100 nM siNC or LAT1 Smartpool siRNA for 72h. Downregulation of LAT1 protein was validated using immunoblotting assay. (**E**) The levels of cytosolic pyruvate, lactate and G-6-P were measured using corresponding kit according to the manufacturer’s instructions. * P ˂ 0.05; ** P ˂ 0.01.

**Fig.S4**


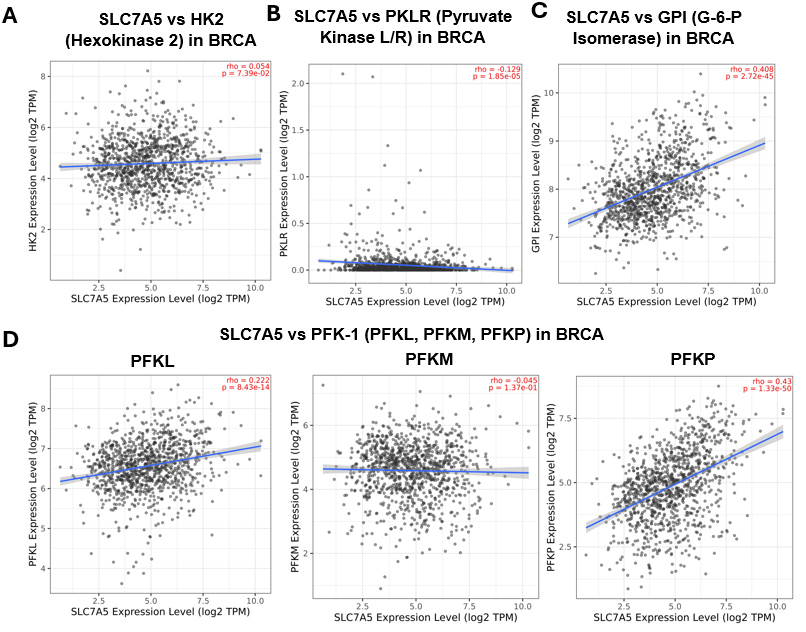


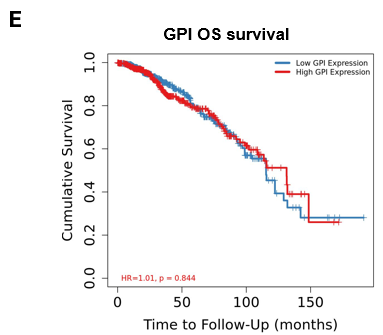


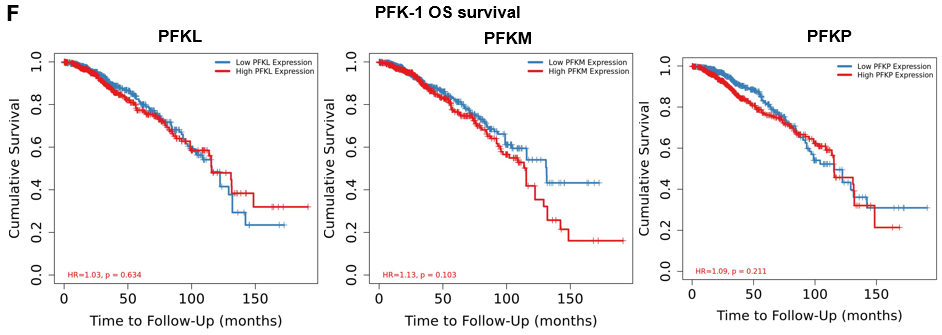


**Suppl Fig. S4.** Gene correlation analysis of SLC7A5 (LAT1) with glycolytic enzymes, including **(A)** HK1, **(B)** PKLR, **(C)** GPI, and **(D)** PFK1 (PFKL, PFKM, PFKP), in breast cancer using the BRCA dataset from TCGA. **(E)** Kaplan-Meier plot of the OS in BC patients from TCGA dataset with high or low expression of GPI using the median value as the cut-off point. **(F)** Kaplan-Meier plot of the OS in BC patients from TCGA dataset with high or low expression of PFKL, PFKM, and PFKP using the median value as the cut-off point.

**Fig.S5**

**A**


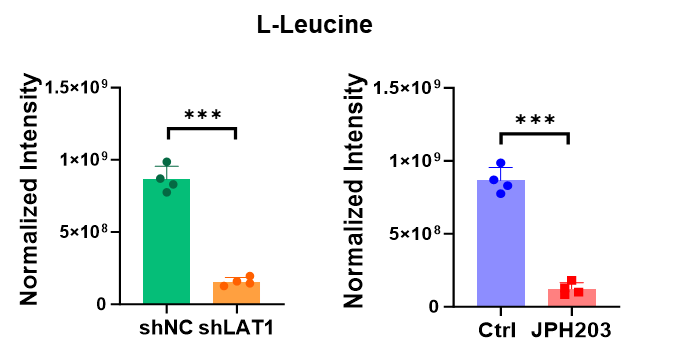


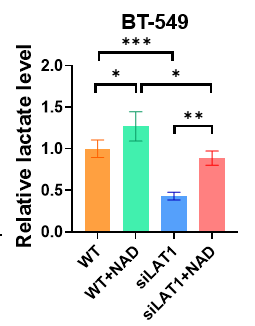
**B**

**C**


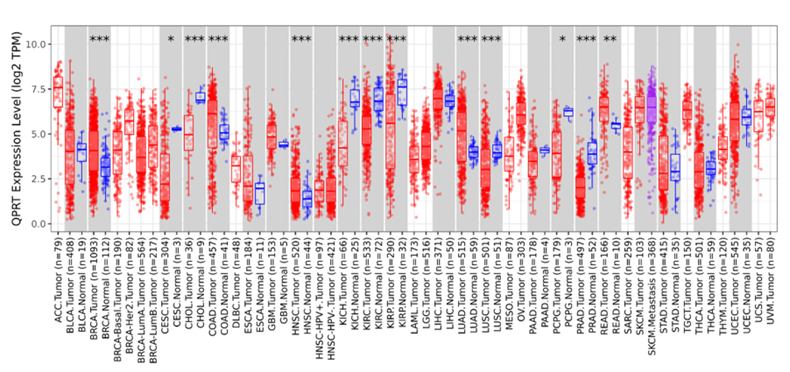


**Suppl Fig. S5**. (**A**) MDA-MB-231 cells were transfected with 100 nM LAT1 Smartpool siRNA or treated with 10 µM JPH203 for 20 min to inhibit the activity of LAT1. LC-MS-based metabolomics was performed to profile changes in cellular metabolites. The concentrations of intracellular L-Leucine in LAT1-inhibited and control cells were measured by metabolomics assay. Peak intensities were normalized for comparison (n = 4). (**B**) BT-549 cells were transiently transfected with 100 nM Smartpool siLAT1 or a negative siRNA control (NC) for 72 h. The lactate levels were measured following the addition of 4mM of NAD+. (**C**) A pan-cancer analysis of QPRT mRNA expression across various cancer types and their corresponding normal tissues using the TCGA database. * P ˂ 0.05; ** P ˂ 0.01; *** P ˂ 0.001.

**Fig. S6**


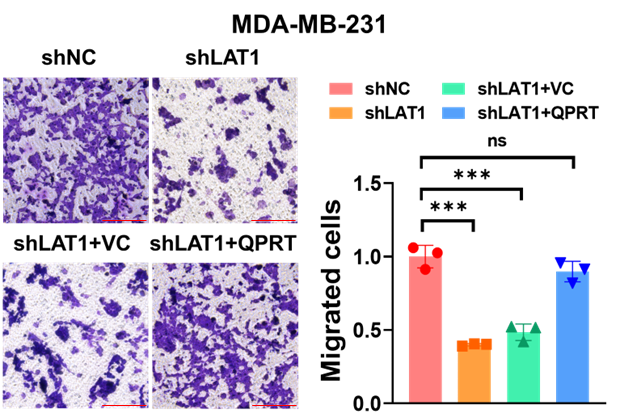


**Suppl Fig. S6**. A transwell migration assay was performed to assess cell migration ability following QPRT OE plasmid and corresponding scamble control transfection in LAT1 shLAT1 and shNC cells. Left: Representative images. Right: Fold change in the number of migrated cells. Scale bar = 200 µm. *** P ˂ 0.001.

**Fig.S7**

**A**

**
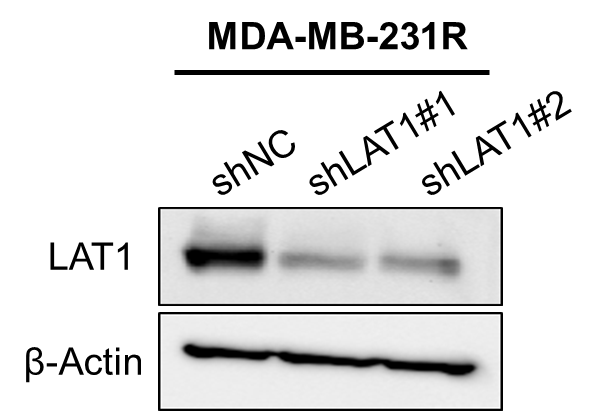
**

**B**


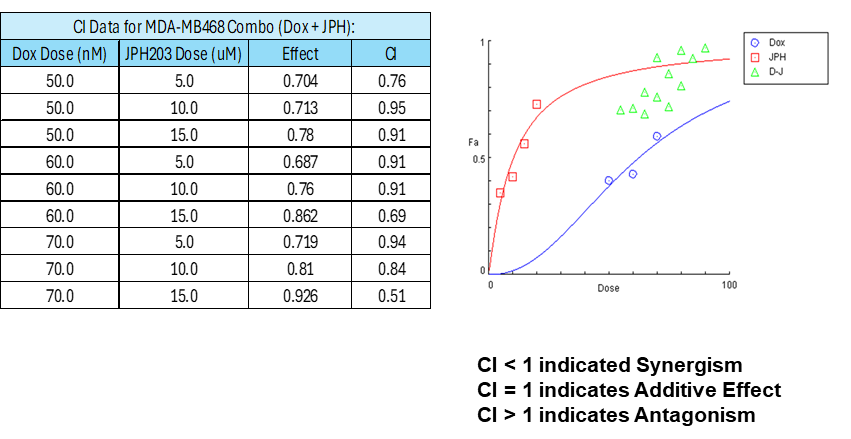


**Suppl Fig. S7.** (**A**) MDA-MB-231R cells were transduced with lentivirus expressing shNC or shLAT1, followed by puromycin selection. LAT1 knockdown was confirmed by Western blot analysis. (**B**) MDA-MB-468 cells were treated with Dox alone, JPH203 alone, or a combination of both drugs at the indicated doses for 72 h. Cell viability was assessed using MTT assays, and combination index (CI) analysis was performed. The figure illustrating the combined effects was generated using CompuSyn software.
